# Genome-wide association study for feed efficiency indicator traits in Nellore cattle considering genotype-by-environment interactions

**DOI:** 10.3389/fgene.2025.1539056

**Published:** 2025-06-02

**Authors:** João B. Silva Neto, Luiz F. Brito, Lucio Flavio M. Mota, Claudio U. Magnabosco, Fernando Baldi

**Affiliations:** ^1^ Department of Animal Science, School of Agricultural and Veterinarian Sciences (FCAV), São Paulo State University (UNESP), Jaboticabal, Brazil; ^2^ Department of Animal Sciences, Purdue University, West Lafayette, IN, United States; ^3^ Cerrados Agricultural Research Center, Embrapa Rice and Beans, Santo Antônio de Goiás, Brazil

**Keywords:** *Bos indicus*, beef cattle, dry matter intake, GWAS, residual feed intake, regulatory pathways

## Abstract

**Introduction:**

Feed efficiency is a key factor in animal production sustainability, directly affecting production costs, environmental efficiency, and farmer profitability. The inclusion of feeding efficiency traits in cattle breeding programs has occurred later than other species due to longer life cycles and the high costs associated with measuring feed intake. However, genomic selection has facilitated the inclusion of difficult-to-measure traits in selection schemes. Thus, understanding the genetic basis of feed efficiency, particularly under varying environmental conditions, is essential.

**Methods:**

This study aimed to identify genomic regions associated with dry matter intake (DMI) and residual feed intake (RFI) in Nellore cattle by performing a genome-wide association study (GWAS) based on single-step genomic reaction norm models that account for genotype-by-environment interactions (G×E). Phenotypic data from 23,170 young bulls and heifers were collected across 301 feed efficiency trials. Genomic windows explaining more than 1% of the total direct additive genetic variance were identified for both the intercept and slope components of the reaction norm for each trait.

**Results:**

For RFI, ten and eleven genomic windows explained more than 1% of the genetic variance for the intercept and slope, respectively. For DMI, 12 windows were identified for the intercept and 17 for the slope. Within these regions, Multiple protein-coding genes were annotated (RFI: 66 for intercept and 47 for slope; DMI: 107 for intercept and 109 for slope), which are involved in key biological processes such as insulin, leptin, glucose, protein, and lipid metabolism; energy balance; heat stress response; feeding behavior; digestion; and nutrient absorption.

**Discussion:**

The results highlight the functional diversity of genes involved in feed efficiency and their dynamic response to environmental variation. While certain genes remained central across environments, others were specifically important under more challenging conditions, emphasizing the role of G×E in regulating these traits. Furthermore, the magnitude and direction of SNP effects varied across environmental gradients, reinforcing the relevance of G×E. Consequently, genomic estimated breeding values for DMI and RFI also differed between environmental extremes. These findings underscore the adaptability of genetic networks to environmental changes and are essential for refining strategies to improve feed efficiency in Nellore cattle.

## 1 Introduction

In animal production, the feed efficiency of individuals is one of the main determinants of production costs, environmental impact, and farm profitability (Nahm, 2002; Kenny et al., 2018). However, despite the strong influence of feed efficiency on the financial return of animal production (Herd and Arthur, 2009; Patience et al., 2015; McLoughlin et al., 2020), in cattle, its measurement and incorporation into selection indices began later compared to poultry and swine (Bottje and Carstens, 2009). This delay can be attributed to several factors unique to poultry and swine farming systems, including shorter life cycles (Tokach et al., 2016; Mottet and Tempio, 2017), easier management, and greater control over environmental and feeding conditions (Kyriazakis, 2011; Gilbert et al., 2015), facilitating standardization and measurement of feed efficiency with greater precision. Furthermore, these species tend to experience greater response to genetic improvement due to the shorter generation intervals and higher selection intensities due to the larger number of offspring per generation.

Given the diversity in cattle production systems and the high costs of accurately measuring individual feed intake, genomic selection (Meuwissen et al., 2001) represents a great opportunity for genetically improving difficult or expensive-to-measure traits such as feed efficiency (Pryce et al., 2014). The wide availability of genomic information has also contributed to a better understanding of the genetic architecture of complex traits, improving the accuracy of selection, particularly for traits with low heritability and more difficult or expensive to measure (Hayes et al., 2007; da Silva Neto et al., 2023), such as feed efficiency traits.

Feed efficiency is influenced by multiple underlying biological mechanisms, such as age, sex, locomotor activity, caloric increment, body composition, feeding behavior, and others (Basarab et al., 2003; Herd et al., 2004; Herd and Arthur, 2009; Haskell et al., 2019). A particularity when considering feed efficiency in ruminants is their ability to convert plant biomass into volatile fatty acids (VFA), proteins, and vitamins due to the presence of microorganisms in the rumen that ferment and transform their feed (McLoughlin et al., 2020; Fregulia et al., 2021; Zhao et al., 2024). These microorganisms are responsible for producing most of the VFAs that serve as metabolizable energy sources for the host (Enjalbert et al., 2017; Zeineldin et al., 2018; McLoughlin et al., 2020; Zhao et al., 2024). Mechanisms related to ruminal function contribute to 23% of the variation in feed efficiency in cattle (Herd et al., 2004). Furthermore, the variability in ruminal microbiota has been associated with feed efficiency, with diet being one of the main components influencing the composition, diversity, and functionality of the rumen microbiome (Krause et al., 2013; Shabat et al., 2016; Ellison et al., 2017; 2019).

Metabolizable energy (ME) is another crucial determinant of feed efficiency in cattle, as it provides the energy needed for vital functions such as maintenance, growth and production (Reynolds et al., 2011; Marcondes et al., 2013; Arndt et al., 2015). Differences in ME utilization efficiency arise from interactions between diet composition, rumen activity and the animal’s physiological processes (Moe, 1981; Reynolds et al., 2011; Hales, 2019). Diets with high energy density, such as those rich in grain, increase ME availability, improving feed efficiency by reducing losses associated with digestion and increasing nutrient assimilation (Reynolds et al., 2011; Hales et al., 2017). In contrast, fiber-rich diets often result in lower ME availability, which poses challenges for animals with higher genetic potential for growth (Nkrumah et al., 2006; Reynolds et al., 2011). Variations in microbiota, driven by diet or environmental factors and management practices, can significantly influence the efficiency of ME utilization (Shabat et al., 2016; Ellison et al., 2017; 2019). These complex interactions between ME, diet, and animal physiology highlight the challenges and opportunities in selecting cattle for greater feed efficiency in diverse production systems.

We have previously assessed genotype-by-environment interactions (G × E) for dry matter intake (DMI) and residual feed intake (RFI) in Nellore cattle using bivariate reaction norm models (RN) (Silva Neto et al., 2023). The environmental gradient (EG) was defined based on the Best Linear Unbiased Estimation (BLUE) solutions of the contemporary groups (CG) for ADG, which captures differences in nutritional, environmental, and management practices during the feed efficiency trials. Heritability estimates for DMI and RFI ranging from 0.26 to 0.54 and 0.07 to 0.41 across EG levels were obtained, respectively, with average genetic correlations for the same trait at different EG of 0.83 and 0.81. The lowest correlations were observed between extreme levels of EG (i.e., 0.22 for RFI and 0.26 for DMI). These results indicated the presence of G × E interactions, particularly under extreme environmental conditions (low and high EG values), resulting in significant reranking of selected animals. These findings underscore the complexities involved in selecting for feed efficiency across varying environments.

Genome-wide association studies have been extensively conducted for traits related to feed efficiency traits in cattle, including DMI and RFI (de Oliveira et al., 2014; Olivieri et al., 2018; Seabury et al., 2017; Brunes et al., 2020). These studies have identified important genetic loci that influence these economically important traits in livestock (Brito et al., 2020). However, there is a notable gap in the literature regarding the inclusion of G × E interactions in these analyses, especially for beef cattle raised under varying environmental conditions, such as in the Nellore breed (Silva Neto et al., 2024). The lack of studies addressing this interaction for traits like DMI and RFI highlights the need for future research that incorporates environmental variation, enabling more precise and effective selection of animals adapted to diverse environmental scenarios.

Therefore, the primary objectives of this study were to: 1) conduct a genome-wide association study (GWAS) using a single-step genomic reaction norm model to identify specific genomic regions associated with dry matter intake and residual feed intake in Nellore cattle (*Bos taurus indicus*) considering G × E interactions; and 2) identify biological processes and metabolic pathways that regulate the expression of DMI and RFI across EG levels. The findings from this study have the potential to provide valuable information into the genetic mechanisms underlying feed efficiency in Nellore cattle, offering a deeper understanding of how environmental conditions modulate the expression of feed efficiency in Nellore cattle.

## 2 Methods

### 2.1 Field data

Individual feed intake was measured on 23,170 Nellore animals (16,430 males and 6,740 females) from 2011 to 2023. The National Association of Breeders and Researchers (ANCP, Ribeirão Preto, SP, Brazil; www.ancp.org.br) provided the data. Animals were recorded during 301 feeding trials and belonged to 25 farms. The dataset used includes phenotypic information for ADG, DMI, and RFI, following the procedures for measuring individual feed intake in beef cattle (Mendes et al., 2020). The herds involved are highly connected due to the use of common sires through artificial insemination (AI), with at least five genetic links across the feeding trials, which were evaluated using the AMC program (Roso and Schenkel, 2006). The animals were raised on pasture-based systems (*Urochloa brizantha cv*). The commercial herds adopted different nutritional practices with some farms providing protein and mineral supplementation, especially during the dry season, while others provided only urea supplementation.

### 2.2 Phenotypic information

The feeding trial was performed in group pens with animals grouped by sex. Feed intake was recorded automatically based using the GrowSafe (www.vytelle.com) and Intergado (www.intergado.com) feeding systems. Detailed information on diet composition, management, and the description of the evaluated traits, i.e., ADG, DMI, and RFI, is provided in Silva Neto et al. (2023). Performance evaluations and feed intake measurements followed the recommended protocols for beef cattle, as described by Mendes et al. (2020). To ensure consistency across trials, it is recommended that the diet be provided *ad libitum* as a total mixed ration (TMR), with a homogeneous blend of forage and concentrate to prevent ingredient selection by the animals. The same standardized dietary formulation should be maintained across all trials conducted at the same facility, with only minimal adjustments in ingredient quantities. Feed refusals should be monitored and maintained between 5% and 10% of the total amount offered. The nutritional value of the diet should reflect that of high-quality pasture, with total digestible nutrient (TDN) levels aligned with the expected average daily weight gain for the animal category under evaluation. The dietary energy concentration should range from 2.4 to 2.8 Mcal of metabolizable energy per kilogram of dry matter, and the average daily gain of the group should not exceed 2.0 kg/day.

Across the feed efficiency trials, variations in dietary composition were observed among different farms, and in some cases, within the same farm across different years. In general, the forage fraction accounted for 70%–80% of the total diet, consisting predominantly of corn or sorghum silage, although some farms used silage from *Brachiaria* grass species. The concentrate fraction primarily consisted of ground corn and ground sorghum, with the addition of protein sources in some trials, such as soybean meal, soybean hulls, and urea.

The dietary effect was indirectly accounted for by including the contemporary group (CG) as a fixed effect in the statistical model (see Section 2.4.1 **)**. Information on geographic regions, climate conditions, and the number of animals per farm is available in Supplementary File 1 (Supplementary Table S1). The descriptive statistics for these traits are reported in Table 1.

**TABLE 1:** Descriptive statistics for dry matter intake (DMI), residual feed intake (RFI), and average daily liveweight gain (ADG) during feeding trials in Nellore cattle.

Variable	RFI (kg/day)	DMI (kg/day)	ADG (kg/day)
Number of phenotypic records (and animals)	23,170	23,170	23,170
Phenotypic average	0.003	8.532	1.231
Standard deviation	0.840	2.153	0.378
Minimum	−7.109	2.519	−0.580
Maximum	6.940	20.658	3.460
Feeding trials information			
Number of trials with only males	211		
Number of trials with only females	90		
Animals in the pedigree	46,631		
Number of sires	2,833		
Number of dams	21,888		
Sires with progeny records	1,024		
Dams with progeny records	11,477		
Number of contemporary groups	760		

### 2.3 Genomic data

A total of 18,567 animals born between 2014 and 2022 were genotyped with a SNP panel containing 65,414 markers (Clarifide^®^ Nelore 3.0). The genotypes were imputed to a SNP panel containing 735,964 markers using the Fimpute 3.0 software (Sargolzaei et al., 2014). The reference population for genotype imputation consisted of 963 representative sires of the main Nellore lineages (i.e., Karvadi, Golias, Godhavari, Taj Mahal, Akasamu, and Nagpur). These reference sires were born between 1995 and 2015 and genotyped with the Illumina BovineHD BeadChip (Illumina Inc., San Diego, CA, USA). Before imputation, we removed non-autosomal markers and autosomal SNPs with GenCall <0.6 to remove genotyping problems. We evaluated imputation accuracy by splitting the reference population into three folds, simulating the medium-density panel density (Mota et al., 2024), resulting in an accuracy of 0.98 like da Silva Neto et al. (2023). The quality control of genotypes after the imputation was performed using the QCF90 software (Misztal et al., 2014). Samples and SNPs with a call rate lower than 0.90 were removed from the dataset. Markers with more than 1% of Mendelian conflicts, with unknown or redundant genomic positions, MAF lower than 0.05, and those located in non-autosomal chromosomes were also removed. After quality control, 18,567 genotyped animals and 452,283 SNPs were retained for further analyses.

### 2.4 Statistical Modelling

#### 2.4.1 Reaction norm models

A two-step reaction norm model (Mota et al., 2020a; Silva Neto et al., 2023) was considered in the present study. In the first step, the ADG during the feeding trials was used to define the EG levels, given that the actual ADG shows significant variation from the recommended ADG of 1.0 kg per day (Mendes et al., 2020). The best linear unbiased estimates (BLUE) solutions of the CG for ADG were used to quantify potential differences between the management, nutritional, and environmental conditions during the feeding trials. Thus, differences in ADG among CG were used as an indirect indicator of better or worse environmental conditions, as higher ADG values were interpreted as being associated with more favorable environments. The CG was defined by year and season of the feeding trial, farm, sex (males and females were allocated to different batches). Age at the beginning of trials (415 ± 116 days of age) was considered a linear covariate in the model. The CG solutions were obtained with an animal model using the best linear unbiased predictions (BLUP) as follows:

y=Xβ+Zα+e

where y represents the phenotypic information for ADG, β is a vector with the fixed effects of CG and age at feeding trails as a linear covariate; α is a vector of additive genetic effects assumed to be normally distributed $N\left(0,A\sigma_{a}^{2}\right)$ , in which $\sigma_{a}^{2}$ is the additive genetic variance and **A** is the pedigree relationship matrix, and **e** is a residual vector assumed $N\left(0,I\sigma_{e}^{2}\right)$ , where **I** is an identity matrix and $\sigma_{e}^{2}$ is the residual variance. **X** and **Z** are incidence matrices linking the records to the fixed and additive genetic effects, respectively. The EG levels were obtained by the BLUE solutions of the CG solutions standardized to a mean value of 0 and standard deviation (SD) of 1.

In the second step, to estimate the GEBV for DMI and RFI across the EG levels, a single step bi-trait genomic reaction norm model (ssBRN) was used as follows:

$$y_{ij}=Xb+\omega_{f}\Phi_{f}\left({EG}_{j}\right)+\alpha_{fi}\Phi_{f}\left({EG}_{j}\right)+e_{ij}$$

where $y_{ij}$ is the vector of phenotypic information for DMI and RFI of the animal *i* recorded at the level *j* of EG, b is the fixed effect of CG and age of animal as linear covariate, X is the incidence matrix, $\omega_{f}$ are the *f-*th fixed regression coefficients (intercept and slope) on $\Phi_{f}\left({EG}_{j}\right)$ ; $\Phi_{f}\left({EG}_{j}\right)$ are the *f-*th Legendre orthogonal polynomials corresponding to *EG* level *j* ( $\left.{EG}_{j}\right)$ , $\alpha_{fi}$ are the random regression coefficients for additive effects of intercept and slope corresponding to animal *i* on EG level *j*, and $e_{ij}$ is a random residual. The ssBRN was fitted considering heterogeneous residual variance across EG levels (Silva Neto et al., 2023).

The additive and residual genetic effects were considered normally distributed: $\alpha =\left\{\alpha \right\}\approx N\left(0,H⊗K\right)$ and $e=\left\{e\right\}\approx N\left(0,I⨂R\right)$ , where K represents the additive genetic variance-covariance matrix attributed to the intercept and slope and R is a diagonal residual variance matrix considering heterogeneous classes; I is an identity matrix, ⊗ is the Kronecker product and H is a matrix combining pedigree and genomic relationship. The inverse $H^{-1}$ was calculated as (Aguilar et al., 2010):

$$H^{-1}=A^{-1}+\left[\begin{array}{cc} 0 & 0\\ 0 & G^{-1}-A_{22}^{-1} \end{array}\right]$$

where, $A^{-1}$ is the inverse of the pedigree-based relationship matrix, $A_{22}^{-1}$ represents the inverse relationship matrix based on pedigree for the genotyped animals, and $G^{-1}$ is the inverse of the genomic relationship matrix obtained according to the first method proposed by VanRaden (2008).

Posterior distribution samples of the (co)variance components were obtained through Bayesian inference using the Gibbs sampling algorithm, implemented in the GIBBSF90 software (Misztal et al., 2014). The Bayesian analyses consisted of a single chain of 500,000 cycles, a burn-in of 50,000 iterations, and storage of values every ten cycles. The convergence was evaluated through visual inspection using the Bayesian Output Analysis (Smith, 2007) and Geweke test (Geweke, 1992).

#### 2.4.2 Estimates of SNPs effects in different environments

The SNP effects for the intercept and slope were obtained using weighted single-step GWAS (WssGWAS) (Wang et al., 2012). The breeding value of the genotyped animals ( $a_{g}$ ) is a function of the SNPs effects:

$$a_{g}=Z_{g}u$$

where $Z_{g}$ represents the incidence matrix of genotypes, and **u** is a vector of the SNPs effects. Thus, the variance of the genetic effects is given by:

$$\text{var }\left(a_{g}\right)=\text{var}\left(Z_{g}u\right)=Z_{g}DZ_{g}´\sigma_{u}^{2}=G*\;\sigma_{a}^{2}$$

where **D** represents the diagonal matrix of the weights for the SNP variances (**D = I** for GBLUP), $\sigma_{u}^{2}$ is the variance of the additive genetic effect obtained from each SNP when the same variance is assumed for all SNPs, $\sigma_{a}^{2}$ is the additive genetic variance of the trait, and G* is the weighted genomic relationship matrix:

$$G*=\frac{\text{var}\;\left(a_{g}\right)}{\sigma_{a}^{2}}=\frac{\text{var}\left(\text{Zu}\right)}{\sigma_{a}^{2}}=Z_{g}DZ_{g}´\lambda$$

where, ***λ*** is a ratio of variances $\left(\frac{\sigma_{u}^{2}}{\sigma_{a}^{2}}\right)$ or normalization constant (Vanraden et al., 2009) . According to Stranden and Garrick (2009), the SNP effects (**û**) can be obtained as follows:

$$û=\frac{\sigma_{u}^{2}}{\sigma_{a}^{2}}\;DZ_{g}´{G*}^{-1}{â}_{g}=DZ_{g}´{\left[Z_{g}DZ_{g}´\right]}^{-1}\;{â}_{g\;}$$

In this way, the best predictor of the SNPs effects given by the genetic effect can be estimated. Estimates of the SNP effects can be used to estimate the individual variance of each SNP effect ( $\sigma_{u,i}^{2}$ ), and apply a different weight to each SNP as follows:

$$\sigma_{u,i}^{2}=u_{i}^{2}2p_{i}\left(1-p_{i}\right)$$

In summary, the SNP effects and weights for the WssGWAS were derived as follows (Wang et al., 2012):

1. Let **D** = **I** in the first step.
2. Calculate G = $Z_{g}DZ_{g}´\lambda$ .
3. Calculate GEBVs for the entire data set using the ssGBLUP.
4. Convert GEBVs to SNP effects (*û*): û = λ **DZ′**( $Z_{g}$ D $Z_{g}$ ′ λ )^−1^ ${â}_{g\;}$ , where ${â}_{g\;}$ is the GEBVs of genotyped animals.
5. Calculate the weight for each SNP: d_i_ = û^2^_i_2p_i_(1−p_i_), where *i* is the *i-th* SNP.
6. Normalize SNP weights to remain the total genetic variance constant.

The SNP weights were calculated iteratively through two iterations. The proportion of the genetic variance explained by moving genomic windows of 100 adjacent SNP were computed according to Wang et al. (2012):

$$\frac{\text{Var}\left(a_{i}\right)}{\sigma_{a}^{2}}\times 100\%=\frac{\text{Var}\left(\sum_{j=1}Z_{j}{\hat{u}}_{j}\right)}{\sigma_{a}^{2}}\times 100\%$$

where $a_{i}$ is the genetic value of the i^th^ region of 100 SNP; $\sigma_{a}^{2}$ is the direct additive genetic variance; Z_j_ is the vector with the genotype of the j^th^ SNP for all animals; and ${\hat{u}}_{j}$ is the estimated effect for the j^th^ SNP within the i-th region. Genomic windows that explained at least 1% of the genetic variance for the slopes were considered potentially associated with animals’ specific responses to changes in EG. The application of SNP windows aims to approximate the structure of haplotype blocks, assuming that these windows can be inherited together (da Silva Neto et al., 2023). The choice of genomic windows consisting of 100 SNPs was based on studies in the literature conducted on Nellore cattle for economic interest traits (Fernandes Júnior et al., 2016; Marín-Garzon et al., 2021; Silva et al., 2024). However, the concept of SNP window has not been unified yet (Garcia et al., 2018).

### 2.5 Gene enrichment analyses

The proportion of the total direct additive genetic variance explained by each genomic window containing 100 SNPs was visualized using Manhattan plots, generated with the CMplot v4.3.0 package in R (Yin et al., 2021). The identified relevant genomic regions were annotated using the *Bos taurus* ARS-UCD1.2 assembly as the reference genome (Rosen et al., 2020). Candidate genes were identified based on the BioMart tool in the ENSEMBL platform (www.ensembl.org/biomart/martview/).

Gene Ontology (GO) and KEGG pathway enrichment analyses (p < 0.05) were conducted using the Database for Annotation, Visualization, and Integrated Discovery (DAVID; version 6.8) (Dennis et al., 2003). This was done to identify biological processes, molecular functions, cellular components, and metabolic pathways associated with positional candidate genes. Interactions between protein-coding genes were predicted using the STRING database with default settings (Szklarczyk et al., 2015).

## 3 Results and discussion

### 3.1 Phenotypic means of RFI, DMI, and ADG across EG levels

The phenotypic means and standard deviations by EG for the studied traits are presented in Table 2. For DMI and ADG, the mean values displayed an increasing trend as the environment became more favorable (or less restrictive), with DMI ranging from 7.14 ± 1.40 (EG 2) to 12.80 ± 3.00 (EG 17) kg of DM/day and ADG from 0.696 (EG 1) to 2.050 (EG 17) kg/day. RFI, an indicator of feed efficiency, remained relatively low and stable across the EG levels, ranging from 0.00 ± 0.687 (EG 2) to 0.30 ± 1.450 (EG 17) kg DM/day. This suggests that, in general, animals consumed more feed and grew faster in better environments, while their efficiency in converting feed to body mass remained largely consistent across EG levels. However, small fluctuations in RFI among EG levels might indicate a slight reduction in feed efficiency under highly favorable environmental conditions. This finding suggests that the animals might consume more feed than needed for growth and maintenance in highly favorable conditions, which could result in less efficient nutrient utilization. Furthermore, the phenotypic expression of RFI can also be influenced by genetic differences in feed utilization, management practices, or dietary composition across farms.

**TABLE 2:** Number of records (N) and descriptive statistics for dry matter intake (DMI), residual feed intake (RFI), and average daily liveweight gain (ADG) by environmental gradient level (EG) in Nellore cattle.

EG	N	DMI (Kg DM/day)	RFI (Kg DM/day)	ADG (Kg/day)
		Mean ± SD		
1	828	7.88 ± 1.76	0.18 ± 0.862	0.696 ± 0.210
2	1,371	7.14 ± 1.40	0.00 ± 0.687	0.839 ± 0.207
3	1,247	7.79 ± 1.77	0.00 ± 0.732	0.936 ± 0.241
4	1,703	7.28 ± 1.39	0.00 ± 0.626	0.960 ± 0.226
5	1,936	7.69 ± 1.42	0.00 ± 0.589	1.040 ± 0.223
6	1,923	7.72 ± 1.38	0.02 ± 0.652	1.100 ± 0.264
7	1,183	7.96 ± 1.69	0.00 ± 0.705	1.140 ± 0.215
8	2,465	8.19 ± 2.10	0.14 ± 1.030	1.210 ± 0.219
9	1,336	8.54 ± 1.69	0.02 ± 0.826	1.270 ± 0.233
10	1,031	8.48 ± 1.93	0.00 ± 0.711	1.300 ± 0.238
11	1,111	8.70 ± 2.02	0.00 ± 0.902	1.320 ± 0.251
12	1,409	9.49 ± 1.92	0.07 ± 0.798	1.410 ± 0.272
13	1,257	9.43 ± 1.84	0.00 ± 0.851	1.430 ± 0.283
14	1,915	9.42 ± 1.94	0.03 ± 0.811	1.490 ± 0.303
15	1,018	9.85 ± 1.82	0.10 ± 0.791	1.630 ± 0.276
16	812	10.80 ± 2.35	0.27 ± 1.380	1.770 ± 0.254
17	625	12.80 ± 3.00	0.30 ± 1.450	2.050 ± 0.377

Considering the recommendation to provide a diet that supports an ADG of around 1.0 kg/day during feeding trials (Mendes et al., 2020), there was significant variability in ADG across the different EGs (Table 2). This variation may be attributed to the physicochemical differences in dietary ingredients, which likely resulted from the wide climatic and geographic diversity across the regions where the trials were held. Another important aspect is that differences in management practices (for example, individual or collective feed distribution systems and different animal densities in the pen), the genetic background of herds, and the genetic selection strategies employed by various farms also play a crucial role in ADG variability. The combination of all these factors underscores the complexity of GxE on feed efficiency traits measured in Nellore cattle (Silva Neto et al., 2023).

### 3.2 Genome-wide association study and functional genomic enrichment

In this study, we performed a GWAS that considered GxE interactions for DMI and RFI, an approach not yet explored in previously published work for these traits. The results are presented in terms of intercept and slope, providing a more detailed assessment of GxE interactions for these feed efficiency traits. The intercept represents the adjusted mean value of the trait, excluding environmental temporal influences (Mota et al., 2020a; b; Silva Neto et al., 2023; 2024). This can be interpreted as the genetic baseline of the trait under idealized conditions, where environmental effects are considered standard. In practice, the intercept captures the genetic variation of the trait before considering interactions with the environment or over time. On the other hand, the slope quantifies the rate of change in the trait as environmental or temporal factors vary (Oliveira et al., 2018; Mota et al., 2020a; b; Silva Neto et al., 2024). The slope measures how the trait responds to these changes, offering insight into the GxE interaction. This model allows for a deeper understanding of the dynamics between genetic and environmental factors in animal performance, aiding in the selection of genetically more adaptable individuals to diverse environmental conditions (Silva Neto et al., 2024).

#### 3.2.1 Intercept for RFI

**FIGURE 1:**
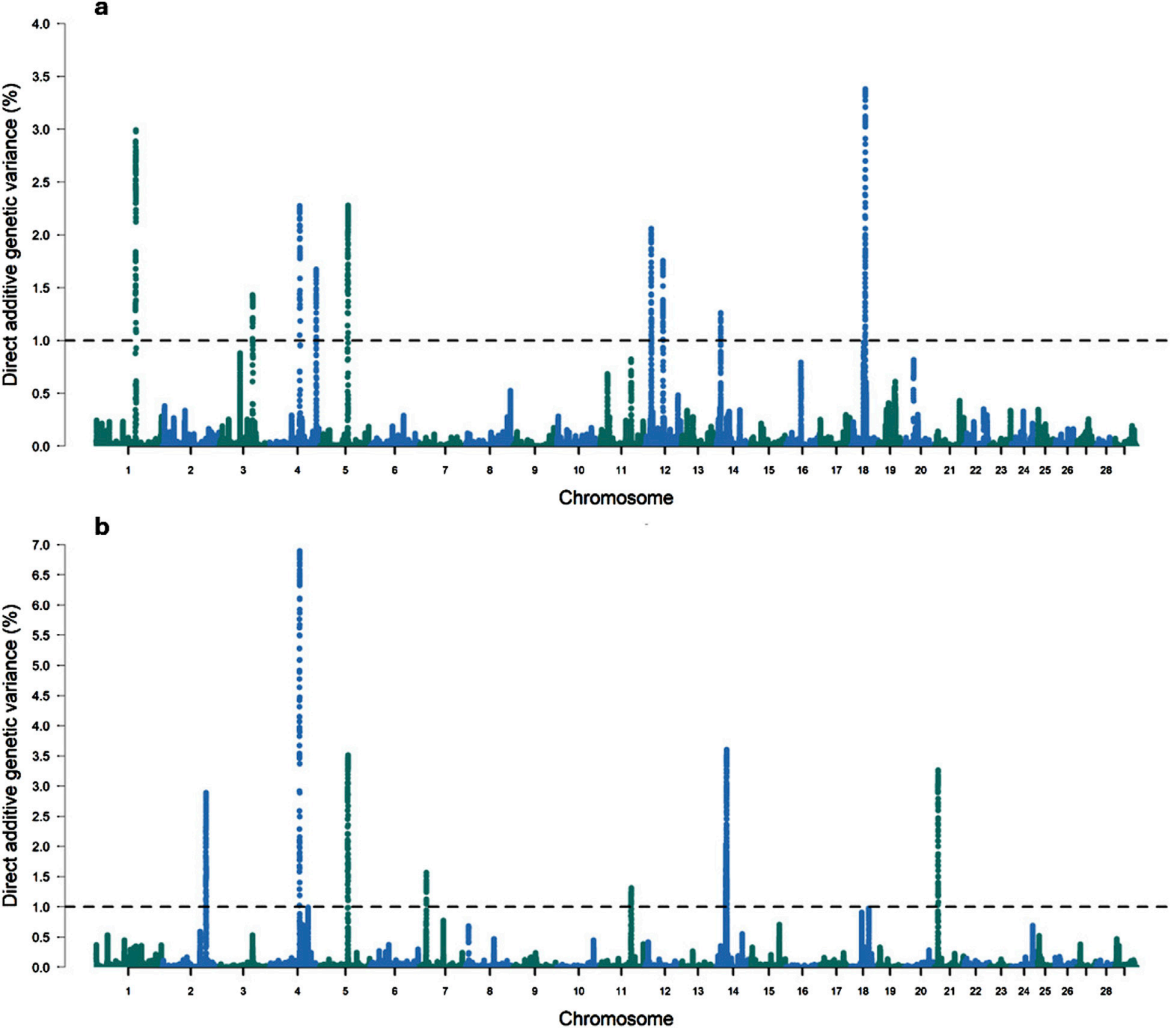
Manhattan plots for the proportion of the total additive genetic variance explained by each genomic window for the intercept **(a)** and slope **(b)** coefficients of the reaction norm model for residual feed intake (RFI) in Nellore cattle. The horizontal line represents the relevance threshold of 1% of the total additive genetic variance explained by each genomic window.

In this study, ten genomic windows explained more than 1% of the intercept’s total direct additive genetic variance for RFI (Table 3; Figure 1). These genomic windows are located on seven chromosomes: BTA1 (94.24–95.04 Mb and 95.05–95.90 Mb), BTA3 (79.33–80.65 Mb), BTA4 (71.07–72.11 Mb and 110.35–110.80 Mb), BTA5 (66.72–67.21 Mb), BTA12 (15.05–15.49 Mb and 42.95–43.49 Mb), BTA14 (10.43–10.64 Mb), and BTA18 (34.60–35.22 Mb). Within these genomic regions, a total of 71 genes were identified, including 2 miRNAs, 66 protein-coding genes, 1 snoRNA, and 2 snRNAs. These results highlight the polygenic architecture of RFI, a trait influenced by multiple genomic regions exerting additive effects on its phenotypic expression.

**TABLE 3:** List of the top genomic windows that explained more than 1% of the total direct additive genetic variance ( $\sigma_{a}^{2}$ ) for residual feed intake (RFI - intercept).

BTA	Location (Mb)	Genes	$\sigma_{a}^{2}$ (%)
18	34.60–35.22	*CDH16, RRAD, CIAO2B, CES2, CES3, CES4A, CBFB, PHAF1, B3GNT9, TRADD, FBXL8, HSF4, NOL3, MATCAP1, EXOC3L1, E2F4, ELMO3, bta-mir-328, TMEM208, FHOD1, SLC9A5, PLEKHG4, KCTD19, LRRC36, TPPP3, ZDHHC1, HSD11B2, ATP6V0D1, AGRP, RIPOR1, CTCF, CARMIL2, ACD, PARD6A, ENKD1, C18H16orf86*	3.41
1	95.05–95.90	*GHSR, FNDC3B, TMEM212, PLD1*	3.02
5	66.72–67.21	*PAH, ASCL1, U1*	2.30
4	71.07–72.11	*GSDME, PALS2, NPY, STK31, FAM221A, ADAM22*	2.29
12	15.05–15.49	*NUFIP1, GPALPP1, GTF2F2, KCTD4, TPT1, SNORA31, SLC25A30*	2.08
12	42.95–43.49*	*-*	1.77
4	110.35–110.80	*CNTNAP2*	1.69
3	79.33–80.65	*LEPR, LEPROT, DNAJC6, AK4, bta-mir-101-1, JAK1, RAVER2, U2*	1.44
1	94.24–95.04	*SPATA16, ECT2, NCEH1, TNFSF10*	1.36
14	10.43–10.64	*ASAP1, CYRIB*	1.27

BTA, *bos taurus* autosome.

Brunes et al. (2020) also identified genomic windows that explained more than 0.5% of the total additive genetic variance for RFI on BTA3 (54.02–54.06 Mb) and BTA5 (70.28–71.12 Mb) in Nellore cattle. Similarly, Olivieri et al. (2016) found regions that explained more than 1.0% of the total additive genetic variance for RFI on BTA1 (100.01–100.02 Mb and 121.63–121.67 Mb), BTA4 (105.90–105.91 Mb and 118.56–118.60 Mb), and BTA18 (11.03–11.06 Mb). Additionally, Bolormaa et al. (2011) identified SNPs significantly associated with RFI on BTA3 (105–106 Mb), BTA4 (41–42Mb and 91–92 Mb), BTA5 (51–52 Mb, 75–76 Mb, 85–86 Mb and 110–111 Mb), BTA12 (55–56 Mb) and BTA18 (3–4 Mb) across seven different cattle breeds (Angus, Murray Grey, Shorthorn, Hereford, Brahman, Santa Gertrudis, and Belmont Red).

Considering the genetic variance explained by the regions that accounted for at least 1% of the direct additive genetic variance, 20.63% of the total direct additive genetic variance was captured. The genomic window on BTA18 (34.60–35.22 Mb) explained the largest proportion of the total additive genetic variance for RFI, accounting for 3.41%, with 36 annotated genes identified within this region (Table 3). The genes located in this genomic window have important functions related to animal performance across different environmental conditions. For instance, the *Cadherin 16* (*CDH16*) gene is a protein primarily expressed in kidney epithelial cells (Lennartz et al., 2023). Cadherins are crucial for cell-cell adhesion, and in the kidney, *CDH16* influences nutrient reabsorption (Igarashi, 2003; Cali et al., 2012; Lennartz et al., 2023). This role is particularly relevant for RFI, as efficient nutrient utilization directly affects the energy balance and intake in cattle (Swanson and Miller, 2008). The *Ras-related glycolysis inhibitor and calcium channel regulator* (*RRAD*) gene is involved in glucose and fatty acid metabolism, which are essential for energy homeostasis (Wang et al., 2014; Lin et al., 2018; Astrain et al., 2022). Its role in regulating glucose levels and insulin signaling could affect how efficiently cattle utilize energy from feed.

Another important set of genes includes *carboxylesterase 2* (*CES2*), *carboxylesterase 3* (*CES3*), and *carboxylesterase 4A* (*CES4A*), which belong to the carboxylesterase family (Hosokawa et al., 2007; Lamego et al., 2013; Liu et al., 2021). These genes are involved in lipid metabolism and detoxification of xenobiotics (Lamego et al., 2013; Liu et al., 2021). The ability of animals to efficiently process and metabolize lipids and other dietary components is particularly important in environments with great variability in feed composition. Differences in carboxylesterase activity could influence how effectively nutrients are converted into energy, thereby affecting feed efficiency (Nawaz et al., 2024). The *heat shock factor 4* (*HSF4*) gene plays a crucial role in cellular responses to heat stress (Lang et al., 2021; Singh et al., 2024). *HSF4* regulates the expression of heat shock proteins, which are important for maintaining protein stability and cellular function under conditions of heat stress (Abbas et al., 2020; Hu et al., 2024; Lang et al., 2021; Tian et al., 2021). In tropical environments, where Nellore cattle are commonly raised, efficient heat shock protein response can preserve metabolic efficiency during hotter conditions. Therefore, genetic variations in the *HSF4* gene may account for differences in cattle responses to heat stress, potentially affecting their feed efficiency and overall energy expenditure.

In the functional enrichment analysis for the intercept of RFI, 17 processes were significantly associated (p-value <0.05) with this trait (Table 4). These processes provide valuable information into the polygenic regulation of RFI and biological mechanisms influencing this trait. One of the biological annotated was the adult feeding behavior (GO:0008343), with the involvement of *growth hormone secretagogue receptor* (*GHSR*), *neuropeptide Y* (*NPY*), and *agouti-related peptide* (*AGRP*) genes. This process directly relates to the regulation of feeding behavior, which is crucial for determining how efficiently an individual converts feed into body mass (Muhammad et al., 2018; Chen et al., 2019). *GHSR* regulates energy balance by mediating the effects of ghrelin, a hormone that stimulates appetite (Klok et al., 2007; Muhammad et al., 2018). *NPY* and *AGRP* are also key regulators of hunger and energy homeostasis (Cansell et al., 2012; Chen et al., 2019). Variations in these genes could result in differences in feed intake and consequently, RFI. Furthermore, positive regulation of appetite (GO:0032100), with the genes *GHSR* and *NPY* (Chen et al., 2019; Zhang et al., 2019), further underscores the relationship between hunger regulation, energy intake, and RFI. In tropical environments, where feed availability and quality may vary, the ability to regulate appetite and energy expenditure becomes critical for maintaining efficient growth and production.

**TABLE 4:** Significant Gene Ontology (GO) terms and Kyoto Encyclopedia of Genes and Genomes (KEGG) pathway analyses for residual feed intake (RFI - intercept).

Category	GO term	Genes symbol	p-value
BP	GO:0008343 - Adult feeding behavior	*GHSR, NPY, AGRP*	<0.001
PWY	bta04920 - Adipocytokine signaling pathway	*TRADD, NPY, LEPR, AGRP*	<0.001
MF	GO:0052689 - Carboxylic ester hydrolase activity	*NCEH1, CES4A, CES2*	0.002
CC	GO:0043231 - Intracellular membrane-bounded organelle	*HSD11B2, DNAJC6, TMEM208, PLD1, CES2*	0.005
BP	GO:0032100 - Positive regulation of appetite	*GHSR, NPY*	0.008
BP	GO:0060259 - Regulation of feeding behavior	*LEPR, AGRP*	0.013
R_PWY	R-BTA-109581 - Apoptosis	*TRADD, TNFSF10, GSDME*	0.016
R_PWY	R-BTA-3371378 - Regulation by c-FLIP	*TRADD, TNFSF10*	0.021
R_PWY	R-BTA-69416 - Dimerization of procaspase-8	*TRADD, TNFSF10*	0.021
PWY	bta04144 - Endocytosis	*PARD6A, DNAJC6, ASAP1, PLD1*	0.022
CC	GO:0005923 - Bicellular tight junction	*PARD6A, PALS2, ECT2*	0.024
R_PWY	KW-0970 - Cilium biogenesis/degradation	*TRADD, TNFSF10*	0.025
BP	R-BTA-140534 - Caspase activation via Death Receptors in the presence of ligand	*KCTD19, KCTD4, ECT2*	0.027
R_PWY	GO:0051260 - Protein homooligomerization	*TRADD, TNFSF10*	0.029
R_PWY	R-BTA-5357769 - Caspase activation via extrinsic apoptotic signalling pathway	*TRADD, TNFSF10, GSDME*	0.030
MF	R-BTA-5357801 - Programmed Cell Death	*DNAJC6, FHOD1, E2F4*	0.031
BP	GO:0019904 - Protein domain specific binding	*GHSR, TPPP3*	0.033

BP, biological process; CC, cellular component; MF, molecular function; PWY, metabolic pathway; R_PWY, biochemical reactions and signaling.

The endocytosis (bta04144) was also annotated in the enrichment analyses, involving *par-6 family cell polarity regulator alpha* (*PARD6A*), *dnaj Heat Shock Protein Family* (HSP40) *Member C6* (*DNAJC6*), *ankyrin repeat and PH domain 1* (*ASAP1*), and *Phospholipase D1* (*PLD1*) genes. This pathway plays a role in cellular nutrient uptake and signaling (Watts and Marsh, 1992; Scita and Fiore, 2010). Endocytosis is essential for internalizing nutrients and cellular receptors (Grant and Donaldson, 2009), which may influence how cattle absorb and process nutrients from their feed, thus impacting feed efficiency. Another important pathway is the adipocytokine signaling pathway (bta04920), involving *TNFRSF1A-Associated via Death Domain* (*TRADD*), *NPY*, *leptin receptor* (*LEPR*), and *AGRP* genes. This pathway plays a major role in energy metabolism and the regulation of fat storage (Jiang et al., 2019; Ahmad et al., 2020). *LEPR* mediates the effects of leptin, a hormone that signals satiety and regulates energy expenditure and fat storage (Meier and Gressner, 2004; Gan et al., 2012; SuárezMesa et al., 2024). Disruptions or variations in this pathway could alter how efficiently cattle utilize energy from feed, influencing their feed efficiency and overall growth (Prihandini et al., 2024).

At the molecular function level, carboxylesterase hydrolase activity (GO:0052689) was also found as a significant process, with genes such as *Neutral cholesterol ester hydrolase 1* (*NCEH1*), *CES4A*, and *CES2* being annotated. Carboxylesterases are enzymes that catalyze the hydrolysis of ester bonds, involved in lipid metabolism and the detoxification of xenobiotics (Lamego et al., 2013; Liu et al., 2021). Efficient lipid metabolism is essential for optimizing energy use, especially under varying environmental conditions where feed quality may differ. This process directly impacts how efficiently cattle convert feed into usable energy, which influences RFI.

#### 3.2.2 Slope for RFI

Eleven relevant genomic windows were identified for the slope of RFI (Table 5; Figure 1). These genomic regions were distributed across seven chromosomes, with four windows located on BTA14 (22.61–22.99 Mb, 22.99–23.45 Mb, 24.39–24.91 Mb, and 24.91–25.43 Mb), two on BTA2 (104.16–104.55 Mb and 104.65–105.41 Mb), and one genomic window each on BTA4 (70.83–71.85 Mb), BTA5 (66.51–67.03 Mb), BTA7 (16.07–16.44 Mb), BTA11 (74.02–74.67 Mb), and BTA21 (7.35–8.15 Mb). These regions overlap with genomic regions previously associated with RFI in Nellore cattle (Mota et al., 2022). These eleven genomic windows accounted for 29.65% of the total direct additive genetic variance for the slope of RFI. In total, 49 genes were mapped within these regions, of which 47 are protein-coding genes and 2 are snRNA genes.

**TABLE 5:** List of the top genomic windows that explained more than 1% of the total direct additive genetic variance ( $\sigma_{a}^{2}$ ) for residual feed intake (RFI - slope).

BTA	Location (Mb)	Genes	$\sigma_{a}^{2}$ (%)
4	70.83–71.85	*OSBPL3, GSDME, PALS2, NPY*	6.89
14	24.39–24.91	*RPL39, UBXN2B, CYP7A1, U1, SDCBP, NSMAF*	3.60
5	66.51–67.03	*U6, PAH, ASCL1, U1*	3.51
21	7.35–8.15	*LRRC28, TTC23, SYNM, IGF1R, PGPEP1L*	3.26
2	104.16–104.55	*XRCC5, MARCHF4, SMARCAL1*	2.89
14	22.99–23.45	*TMEM68, TGS1, LYN, RPS20, U1, MOS, PLAG1, CHCHD7, SDR16C5, FAM110B*	2.04
2	104.65–105.41	*IGFBP5, TNP1*	1.65
7	16.07–16.44	*INSR, ARHGEF18, PEX11G, TEX45, ZNF358, MCOLN1, PNPLA6*	1.56
14	24.91–25.43	*TOX*	1.49
14	22.61–22.99	*XKR4*	1.45
11	74.02–74.67	*DNMT3A, POMC, EFR3B, DNAJC27, ADCY3, CENPO, PTRHD1, NCOA1*	1.31

BTA: bos taurus autosome

The genomic window located on BTA4 (70.83–71.85 Mb) explained the largest proportion of the total additive genetic variance for the slope of RFI (6.89%), and the *Oxysterol Binding Protein-Like 3* (*OSBPL3*), *Gasdermin E* (*GSDME*), *Protein Associated with Lin Seven 2* (*PALS2*), and *NPY* genes were identified within this window. *OSBPL3* is involved in lipid metabolism and intracellular lipid transport, suggesting a potential effect in energy homeostasis and efficiency (Song et al., 2012), which are processes that have an influence in RFI. *GSDME* is known for its role in programmed cell death (pyroptosis), which could influence energy expenditure through tissue turnover and inflammatory responses, thereby impacting metabolic efficiency (Zhu et al., 2024). *NPY* is a key regulator of appetite and energy balance, making it a direct candidate for influencing feed intake and energy utilization (Cansell et al., 2012; Chen et al., 2019). The involvement of these genes in some metabolic pathways related to energy balance and tissue homeostasis could explain their contribution to the genetic variance in the slope of RFI. This suggests that variations in the expression or function of these genes might modulate how Nellore cattle adjust their feed intake and energy expenditure in response to environmental or nutritional changes, thereby affecting the slope of the reaction norm for RFI.

The functional enrichment analyses of genes found in the genomic windows that explained the largest proportion of the genetic variance of the slope of RFI in Nellore cattle are displayed in Table 6. Twenty-nine mechanisms were significantly associated with the slope of RFI (p-value <0.05), including the positive regulation of the MAPK cascade (GO:0043410), which plays a crucial role in mediating cellular responses to environmental stimuli (Bardwell, 2006; Meng and Zhang, 2013). The MAPK signaling pathway influences growth, cell proliferation, and stress response, essential for maintaining metabolic balance in varying environmental conditions (Bardwell, 2006; Meng and Zhang, 2013; Yue and López, 2020). The *v-mos Moloney murine sarcoma viral oncogene homolog* (*MOS)*, *LYN proto-oncogene*, *Src family tyrosine kinase (LYN)*, *Insulin receptor (INSR)*, *Gasdermin E (GSDME)*, and *Insulin-like growth factor 1 receptor (IGF1R)* genes were annotated and involved in the positive regulation of the MAPK cascade (Gonzalez-Garcia et al., 2014; Werner, 2023). *MOS* is a key regulator of the MAPK pathway, primarily known for its role in cellular proliferation and differentiation (Gonzalez-Garcia et al., 2014). Changes in the regulation of the MAPK pathway by *MOS* could alter energy balance and metabolic rate (Okazaki and Sagata, 1995; Adhikari and Cullen, 2014), which are critical for feed efficiency. *LYN* plays a significant role in the activation of the MAPK pathway (Avila et al., 2012), and variations in the expression of *LYN* may influence how cattle respond to feeding under different environmental conditions, possibly by affecting energy expenditure and metabolic adjustments. The *LYN* gene was also found in a GWAS study for growth traits in Nellore cattle (Terakado et al., 2018). The *INSR* gene is associated with the insulin signaling pathway, closely interacting with the MAPK cascade (Zhang et al., 2011; Werner, 2023). Insulin is a key regulator of glucose metabolism and energy homeostasis (Payankaulam et al., 2019). Genetic variation in *INSR* may influence how cattle manage nutrient absorption, storage, and overall energy balance. Given the importance of glucose metabolism on efficient feed use, *INSR* variants could impact feed efficiency by modulating energy use under different environmental conditions, thus influencing RFI. Alongside *INSR*, the *IGF1R* gene also plays a pivotal role in growth, development, and nutrient partitioning, all of which are integral to feed efficiency (Yang et al., 2019; Mota et al., 2022). The interaction of *IGF1R* with the MAPK pathway underlines its importance in mediating growth and metabolic responses, particularly in response to environmental changes (Yang et al., 2019). Mutations in *IGF1R* may alter the cattle’s ability to utilize feed for efficient growth, affecting how well animals adapt their nutrient use in response to varying environmental conditions.

**TABLE 6:** Significant Gene Ontology (GO) terms and Kyoto Encyclopedia of Genes and Genomes (KEGG) pathway analyses for residual feed intake (RFI - slope).

Category	GO term	Gene symbol	p-value
BP	GO:0043410 - Positive regulation of MAPK cascade	*MOS, LYN, INSR, GSDME, IGF1R*	<0.001
MF	GO:0031994 - Insulin-like growth factor I binding	*IGFBP5, INSR, IGF1R*	<0.001
R_PWY	R-BTA-211976 - Endogenous sterols	*NCOA1, POMC, CYP7A1*	0.001
R_PWY	R-BTA-8957322 - Metabolism of steroids	*NCOA1, POMC, OSBPL3, CYP7A1*	0.001
R_PWY	R-BTA-192105 - Synthesis of bile acids and bile salts	*NCOA1, OSBPL3, CYP7A1*	0.001
R_PWY	R-BTA-194068 - Bile acid and bile salt metabolism	*NCOA1, OSBPL3, CYP7A1*	0.002
R_PWY	R-BTA-556833 - Metabolism of lipids	*NCOA1, POMC, OSBPL3, TGS1, PNPLA6, CYP7A1*	0.003
R_PWY	R-BTA-211897 - Cytochrome P450 - Arranged by substrate type	*NCOA1, POMC, CYP7A1*	0.003
CC	GO:0005899 - Insulin receptor complex	*INSR, IGF1R*	0.006
MF	GO:0005009 - Insulin receptor activity	*INSR, IGF1R*	0.006
PWY	bta04923 - Regulation of lipolysis in adipocytes	*INSR, NPY, ADCY3*	0.007
PWY	bta04213 - Longevity regulating pathway - multiple species	*INSR, ADCY3, IGF1R*	0.007
MF	GO:0043559 - Insulin binding	*INSR, IGF1R*	0.008
PWY	bta04913 - Ovarian steroidogenesis	*INSR, ADCY3, IGF1R*	0.008
R_PWY	R-BTA-211945 - Phase I - Functionalization of compounds	*NCOA1, POMC, CYP7A1*	0.009
MF	GO:0031995 - Insulin-like growth factor II binding	*IGFBP5, INSR*	0.014
MF	GO:0043560 - Insulin receptor substrate binding	*INSR, IGF1R*	0.016
PWY	bta04211 - Longevity regulating pathway	*INSR, ADCY3, IGF1R*	0.016
PWY	bta04914 - Progesterone-mediated oocyte maturation	*MOS, ADCY3, IGF1R*	0.017
R_PWY	R-BTA-193807 - Synthesis of bile acids and bile salts via 27-hydroxycholesterol	*NCOA1, CYP7A1*	0.020
MF	GO:0043548 - Phosphatidylinositol 3-kinase binding	*INSR, IGF1R*	0.025
R_PWY	R-BTA-193368 - Synthesis of bile acids and bile salts via 7alpha-hydroxycholesterol	*NCOA1, CYP7A1*	0.028
BP	GO:0030335 - Positive regulation of cell migration	*LYN, INSR, IGF1R*	0.028
PWY	bta04114 - Oocyte meiosis	*MOS, ADCY3, IGF1R*	0.030
BP	GO:0071333 - Cellular response to glucose stimulus	*CYP7A1, IGF1R*	0.035
PWY	bta04915 - Estrogen signaling pathway	*NCOA1, POMC, ADCY3*	0.035
R_PWY	R-BTA-211859 - Biological oxidations	*NCOA1, POMC, CYP7A1*	0.036
R_PWY	R-BTA-400206 - Regulation of lipid metabolism by PPARalpha	*NCOA1, TGS1*	0.036
MF	GO:0005184 - Neuropeptide hormone activity	*POMC, NPY*	0.048

BP, biological process; CC, cellular process; MF, molecular function; PWY, metabolic pathaway; R_PWY, biochemical reactions and signaling.

Other biological processes associated with *IGF1R* and *INSR,* including insulin-like growth factor I binding (GO:0031994), insulin receptor activity (GO:0005158), and insulin receptor complex (GO:0005899) were found in the enrichment analysis for the slope of RFI. These processes are crucial for regulating energy balance and nutrient partitioning. Insulin-like growth factor I (IGF-I) is critical for growth and metabolic regulation, with its binding modulating activity in pathways central to nutrient efficiency (Pereira et al., 2016; Rieger and O’Connor, 2021; Díaz Del Moral et al., 2022). More efficient insulin receptor activity could allow cattle to optimize energy use, particularly in response to environmental challenges, ensuring consistent feed efficiency. This pathway may be important in determining how well animals adapt their nutrient utilization strategies in response to GxE interactions. The slope of RFI, which reflects the animal’s efficiency in utilizing feed under different conditions, could thus be significantly influenced by the genetic variation within the insulin and IGF signaling pathways.

Enriching pathways related to bile acid and salt metabolism (R-BTA-194068) further emphasizes the importance of lipid homeostasis in determining feed efficiency. Bile acids are essential for fat digestion and absorption, and the *cytochrome P450 family 7 subfamily A member 1* (*CYP7A1*) and *nuclear receptor coactivator 1* (*NCOA1*) genes are key regulators of bile acid metabolism (Jia et al., 2024). Variations in the efficiency of bile acid metabolism could influence the absorption of nutrients (Jia et al., 2021), particularly lipids, which are critical for energy balance. Animals with optimized bile acid metabolism may be better able to maintain feed efficiency under fluctuating environmental conditions, contributing to differences in the RFI slope.

The neuropeptide hormone activity (GO:0005184) was also one of the processes significatively associated with RFI. The ability of animals to regulate feed intake through neuroendocrine mechanisms may be a key determinant of how efficiently they convert feed into body mass, particularly when facing environmental variability. The *pro-opiomelanocortin* (*POMC*) and *adrenocorticotropic hormone* (*ACTH*) genes play fundamental roles in appetite regulation and energy balance (Millington, 2007; Hasenmajer et al., 2021). This regulation could explain variations in feed efficiency as environmental conditions change, influencing the slope of RFI.

The regulation of lipid metabolism by PPARalpha (R-BTA-400206) pathway, which includes *Nuclear Receptor Coactivator 1* (*NCOA1*) and *Trimethylguanosine Synthase* (*TGS1*), highlights the role of lipid metabolism in RFI expression. PPARalpha (peroxisome proliferator-activated receptor alpha) is a critical regulator of lipid metabolism, particularly in response to fasting or limited nutrient availability (Lefebvre et al., 2006; Bougarne et al., 2018; Fuior et al., 2023). This pathway may influence how animals utilize lipids for energy under stressful or nutrient-limited conditions, which could affect the slope of RFI by enabling animals to maintain energy balance and feed efficiency across different environments.

#### 3.2.3 Intercept for DMI

**FIGURE 2:**
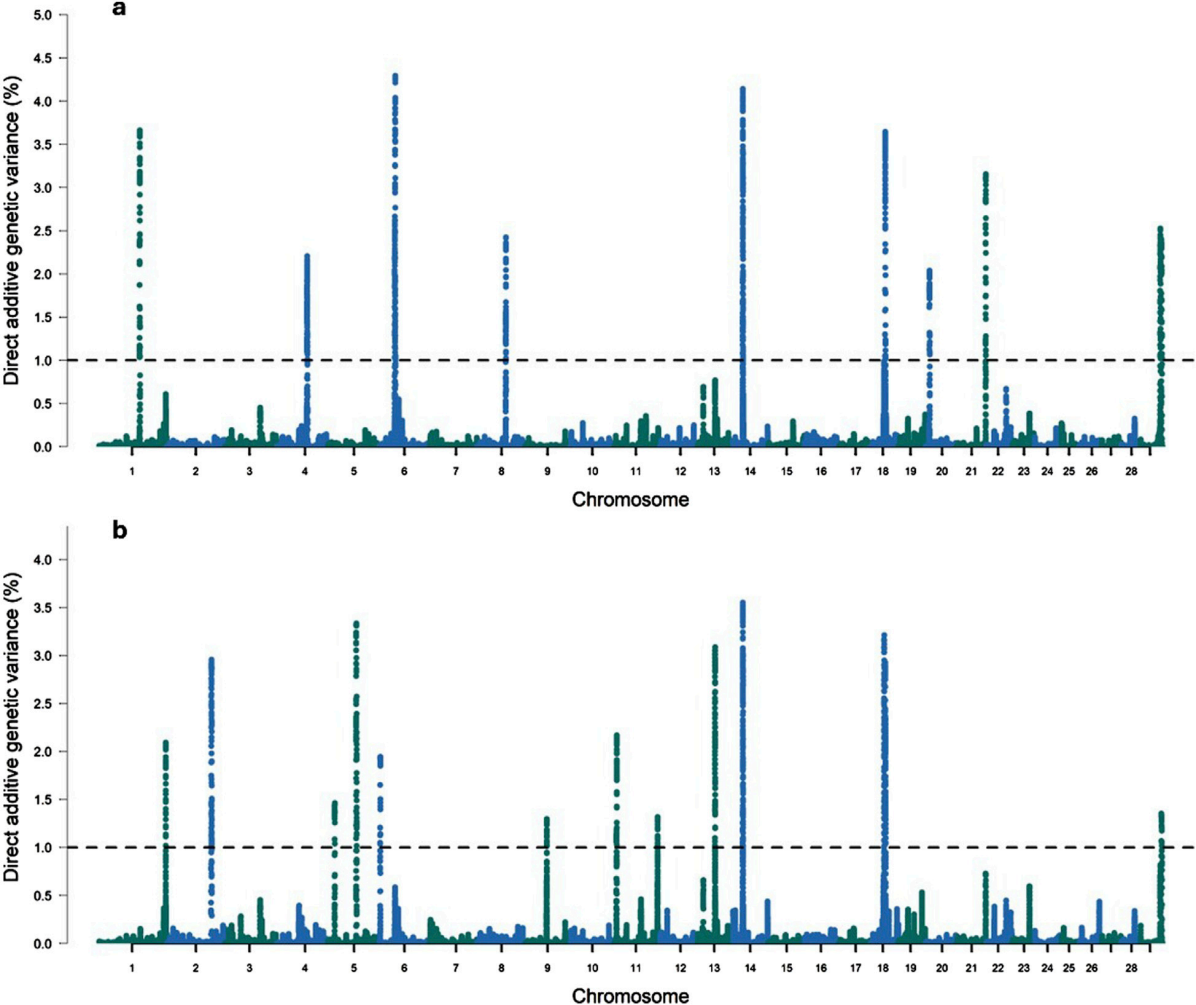
Manhattan plots for the proportion of the total additive genetic variance explained by each genomic window for the intercept **(a)** and slope **(b)** coefficients of the reaction norm model for dry matter intake (DMI) in Nellore cattle. The horizontal line represents the relevance threshold of 1% of the total additive genetic variance explained by each genomic window.

Twelve genomic windows explaining more than 1% of the total direct additive genetic variance of the intercept for DMI were identified as shown in Table 7 and Figure 2. These genomic regions are located on seven chromosomes: BTA1 (95.09–95.95), BTA4 (70.88–71.88 Mb), BTA6 (36.02–36.57 Mb and 37.15–37.95 Mb), BTA8 (67.24–67.72 Mb), BTA14 (22.90–23.31 Mb and 23.33–23.89 Mb), BTA18 (34.83–35.42 Mb), BTA20 (9.15–9.83 Mb), BTA21 (68.47–68.77 Mb) and BTA29 (46.18–47.10 Mb and 48.54–50.15 Mb). Some regions in these chromosomes were also identified as associated with DMI in other GWAS studies with cattle (Serão et al., 2013; de Oliveira et al., 2014; Brunes et al., 2020; Mota et al., 2022). A total of 112 genes were identified within these genomic windows, including 107 protein-coding genes, three miRNAs, and two snRNAs. These findings highlight the polygenic nature of DMI, a trait influenced by numerous genomic regions that collectively contribute to its phenotypic expression, as RFI.

**TABLE 7:** List of the top genomic windows that explained more than 1% of the total direct additive genetic variance ( $\sigma_{a}^{2}$ ) for dry matter intake (DMI - intercept).

BTA	Location (Mb)	Genes	$\sigma_{a}^{2}$ (%)
6	37.15–37.95	*LAP3, MED28, FAM184B, DCAF16, NCAPG, LCORL*	4.29
14	22.90–23.31	*XKR4, TMEM68, TGS1, LYN, RPS20, U1, MOS*	4.14
1	95.09–95.95	*FNDC3B, TMEM212, PLD1*	3.66
18	34.83–35.42	*E2F4, ELMO3, bta-mir-328, TMEM208, FHOD1, SLC9A5, PLEKHG4, KCTD19, LRRC36, TPPP3, ZDHHC1, HSD11B2, ATP6V0D1, AGRP, RIPOR1, CTCF, CARMIL2, ACD, PARD6A, ENKD1, C18H16orf86, GFOD2, RANBP10, TSNAXIP1, CENPT, THAP11, NUTF2, EDC4, NRN1L, PSKH1, PSMB10*	3.64
21	68.47–68.77	*TDRD9, RD3L, ASPG, MIR203B, KIF26A*	3.15
6	36.02–36.57	*FAM13A, HERC3, NAP1L5, PYURF, HERC5, HERC6, PPM1K, ABCG2, U6, bta-mir-10170*	2.66
29	46.18–47.10	*CPT1A, MRPL21, IGHMBP2, MRGPRF, TPCN2, CCND1, LTO1, FGF19, FGF4, FGF3*	2.52
8	67.24–67.72	*SLC18A1, ATP6V1B2, LZTS1*	2.42
29	48.54–50.15	*U6, CARS1, NAP1L4, PHLDA2, SLC22A18, CDKN1C, KCNQ1, TRPM5, TSSC4, TSPAN32, ASCL2, TH, INS, IGF2, TNNT3, LSP1, PRR33, TNNI2, SYT8, CTSD, IFITM10, DUSP8, MOB2, BRSK2*	2.40
4	70.88–71.88	*OSBPL3, GSDME, PALS2, NPY*	2.21
20	9.15–9.83	*ZNF366, PTCD2, MRPS27, MAP1B*	2.04
14	23.33–23.89	*PLAG1, CHCHD7, SDR16C5, SDR16C6, PENK, U6, BPNT2*	1.70

BTA, *bos taurus* autosome.

The relevant genomic regions for DMI explained 34.83% of the overall direct additive genetic variance. The genomic window located on BTA6 (37.15–37.95 Mb) explained the largest portion of additive genetic variance, accounting for 4.29%, with six annotated genes identified within this region (Table 7). The *Leucine Aminopeptidase 3* (*LAP3*) is involved in protein degradation, processing and regulating peptide breakdown (Yao et al., 2021; Wang et al., 2024). It has been associated with growth traits in Holstein cattle and Yak (Bos grunniens) (Yao et al., 2019; Wang et al., 2024). Given its role in protein metabolism, *LAP3* may influence the efficiency of cattle utilizing nutrients, which is directly related to DMI. Therefore, efficient protein metabolism could enable animals to optimize their intake for growth and maintenance under varying environmental conditions. Another important gene widely associated with growth traits and feed intake in cattle is *Non-SMC Condensin I Complex Subunit G* (*NCAPG)* (Hoshiba et al., 2013; Lindholm-Perry et al., 2013). Studies have shown that polymorphisms in *NCAPG* are linked to ADG and DMI in cattle (Angus, Hereford, Simmental, Limousin, Cha rolais, Gelbvieh and Red Angus) (Lindholm-Perry et al., 2011; Lindholm-Perry et al., 2013; Seabury et al., 2017). This gene is involved in cell cycle regulation and it has been associated with growth rate and body size in several cattle breeds (Setoguchi et al., 2011; Zhang et al., 2016). *NCAPG* influences feed intake by modulating growth demands, where larger or faster-growing animals require more feed to meet their energy needs. This makes it a strong candidate gene for influencing DMI in response to average environmental conditions.

The *Ligand Dependent Nuclear Receptor Corepressor Like* (*LCORL*) is a transcription factor associated with skeletal growth and body size in humans, horses, and cattle (Utsunomiya et al., 2013; Al-Mamun et al., 2015). *LCORL* has been linked to growth traits and feed efficiency in cattle, often acting in concert with *NCAPG* (La et al., 2019). Polymorphisms in *LCORL* have been correlated with feed intake and gain, particularly in beef cattle (Angus, Hereford, Simmental, Limousin, Cha rolais, Gelbvieh and Red Angus) (Lindholm-Perry et al., 2013). Its role in skeletal growth may be crucial for determining body size and the corresponding feed requirements, thereby influencing DMI. The *DDB1 and CUL4 Associated Factor 16* (*DCAF16*) is part of the ubiquitin-proteasome pathway, essential for protein degradation and cellular homeostasis (Zhang et al., 2021). By influencing protein degradation, it indirectly impacts growth rate and metabolic efficiency (Mistry et al., 2020; Zhang et al., 2021), potentially altering the energy requirements and feed intake of cattle. Since protein metabolism is energy-intensive, variations in this gene may affect how animals convert feed into growth.

In the functional enrichment analysis, 53 processes were found to be significantly associated (p-value <0.05) with the intercept of DMI (Table 8). These processes offer important insights into the polygenic control of DMI and the biological pathways affecting its regulation. In comparison to the significant processes identified for RFI, DMI had a greater number of associated processes. This can be attributed to the larger number of relevant genomic windows identified for DMI, which also reflected in a higher number of genes found and involved in regulating this trait.

**TABLE 8:** Significant Gene Ontology (GO) terms and Kyoto Encyclopedia of Genes and Genomes (KEGG) pathway analyses for dry matter intake (DMI - intercept).

Category	GO term	Gene symbol	p-value
R_PWY	R-BTA-74752 - Signaling by Insulin receptor	*FGF19, ATP6V1B2, ATP6V0D1, CTSD, FGF3, FGF4, INS*	<0.001
R_PWY	R-BTA-9006934 - Signaling by Receptor Tyrosine Kinases	*FGF19, ATP6V1B2, IGF2, ATP6V0D1, CTSD, FGF3, FGF4, INS*	<0.001
R_PWY	R-BTA-77387 - Insulin receptor recycling	*ATP6V1B2, ATP6V0D1, CTSD, INS*	<0.001
BP	GO:0009887 - Animal organ morphogenesis	*FGF19, E2F4, PHLDA2, FGF3, FGF4*	<0.001
R_PWY	R-BTA-2428928 - IRS-related events triggered by IGF1R	*FGF19, IGF2, FGF3, FGF4*	<0.001
R_PWY	R-BTA-2428924 - IGF1R signaling cascade	*FGF19, IGF2, FGF3, FGF4*	<0.001
R_PWY	R-BTA-2404192 - Signaling by Type 1 Insulin-like Growth Factor 1 Receptor (IGF1R)	*FGF19, IGF2, FGF3, FGF4*	<0.001
R_PWY	R-BTA-74751 - Insulin receptor signalling cascade	*FGF19, FGF3, FGF4, INS*	<0.001
BP	GO:0043410 - Positive regulation of MAPK cascade	*MOS, LYN, IGF2, GSDME, INS*	0.001
CC	GO:0005737 - Cytoplasm	*RIPOR1, BRSK2, TSSC4, FHOD1, IGHMBP2, NCAPG, FGF3, FGF4, HERC5, HERC3, CCND1, TSNAXIP1, E2F4, PHLDA2, LZTS1, HERC6, LYN, TDRD9, DUSP8, NAP1L4, THAP11, MED28, MOS, TPPP3, TH, KIF26A, FGF19, MOB2, RANBP10, ELMO3, LAP3, CARS1, GSDME*	0.001
BP	GO:0001934 - Positive regulation of protein phosphorylation	*CCND1, FGF19, MOB2, FGF3, FGF4*	0.002
R_PWY	R-BTA-1257604 - PIP3 activates AKT signaling	*FGF19, FGF3, PSMB10, FGF4, INS*	0.003
R_PWY	R-BTA-199418 - Negative regulation of the PI3K/AKT network	*FGF19, FGF3, FGF4, INS*	0.003
R_PWY	R-BTA-6811558 - PI5P, PP2A and IER3 Regulate PI3K/AKT Signaling	*FGF19, FGF3, FGF4, INS*	0.003
R_PWY	R-BTA-9006925 - Intracellular signaling by second messengers	*FGF19, FGF3, PSMB10, FGF4, INS*	0.004
MF	GO:0005104 - Fibroblast growth factor receptor binding	*FGF19, FGF3, FGF4*	0.005
BP	GO:0010628 - Positive regulation of gene expression	*PLAG1, FGF19, CTCF, FGF3, FGF4, INS*	0.005
PWY	bta05218 - Melanoma	*CCND1, FGF19, FGF3, FGF4*	0.005
PWY	bta04014 - Ras signaling pathway	*FGF19, IGF2, PLD1, FGF3, FGF4, INS*	0.006
BP	GO:0030334 - Regulation of cell migration	*FGF19, PHLDA2, FGF3, FGF4*	0.007
R_PWY	R-BTA-109704 - PI3K Cascade	*FGF19, FGF3, FGF4*	0.007
R_PWY	R-BTA-112399 - IRS-mediated signalling	*FGF19, FGF3, FGF4*	0.008
MF	GO:0003779 - actin binding	*MAP1B, TNNT3, TNNI2, LSP1, MED28*	0.008
BP	GO:0051781 - Positive regulation of cell division	*IGF2, FGF3, FGF4*	0.009
CC	GO:0045121 - Membrane raft	*LYN, KCNQ1, CTSD, ABCG2*	0.014
PWY	bta04010 - MAPK signaling pathway	*FGF19, IGF2, DUSP8, FGF3, FGF4, INS*	0.014
BP	GO:0006006 - Glucose metabolic process	*KCNQ1, IGF2, INS*	0.015
R_PWY	R-BTA-917937 - Iron uptake and transport	*ATP6V1B2, ATP6V0D1, ABCG2*	0.016
BP	GO:0008543 - Fibroblast growth factor receptor signaling pathway	*FGF19, FGF3, FGF4*	0.016
R_PWY	R-BTA-162582 - Signal Transduction	*CPT1A, FGF19, NPY, PENK, ATP6V1B2, IGF2, ATP6V0D1, CTSD, FGF3, PSMB10, FGF4, INS*	0.019
PWY	bta04015 - Rap1 signaling pathway	*PARD6A, FGF19, FGF3, FGF4, INS*	0.020
MF	GO:0008083 - Growth factor activity	*FGF19, IGF2, FGF3, FGF4*	0.025
PWY	bta04810 - Regulation of actin cytoskeleton	*MOS, FGF19, FGF3, FGF4, INS*	0.025
BP	GO:0008343 - Adult feeding behavior	*NPY, AGRP*	0.026
R_PWY	R-BTA-190236 - Signaling by FGFR	*FGF19, FGF3, FGF4*	0.026
R_PWY	R-BTA-5658623 - FGFRL1 modulation of FGFR1 signaling	*FGF3, FGF4*	0.026
R_PWY	R-BTA-382551 - Transport of small molecules	*SLC9A5, SLC22A18, ATP6V1B2, ATP6V0D1, PSMB10, ABCG2*	0.031
CC	GO:0016324 - Apical plasma membrane	*PARD6A, KCNQ1, ATP6V1B2, PLD1, ABCG2*	0.032
CC	GO:0005861 - Troponin complex	*TNNT3, TNNI2*	0.035
PWY	bta05224 - Breast cancer	*CCND1, FGF19, FGF3, FGF4*	0.036
BP	GO:1902600 - Proton transmembrane transport	*SLC9A5, ATP6V1B2, ATP6V0D1*	0.037
PWY	bta05226 - Gastric cancer	*CCND1, FGF19, FGF3, FGF4*	0.038
BP	GO:0046628 - Positive regulation of insulin receptor signaling pathway	*IGF2, INS*	0.038
PWY	bta04151 - PI3K-Akt signaling pathway	*CCND1, FGF19, IGF2, FGF3, FGF4, INS*	0.039
BP	GO:0007218 - Neuropeptide signaling pathway	*NPY, PENK, AGRP*	0.043
MF	GO:0005159 - Insulin-like growth factor receptor binding	*IGF2, INS*	0.043
R_PWY	R-BTA-5683057 - MAPK family signaling cascades	*FGF19, FGF3, PSMB10, FGF4*	0.046
R_PWY	R-BTA-5654228 - Phospholipase C-mediated cascade; FGFR4	*FGF19, FGF4*	0.047
R_PWY	R-BTA-5654219 - Phospholipase C-mediated cascade: FGFR1	*FGF3, FGF4*	0.047
R_PWY	R-BTA-190242 - FGFR1 ligand binding and activation	*FGF3, FGF4*	0.047
R_PWY	R-BTA-190322 - FGFR4 ligand binding and activation	*FGF19, FGF4*	0.047
CC	GO:0030672 - Synaptic vesicle membrane	*ATP6V1B2, SYT8, SLC18A1*	0.048
PWY	bta04920 - Adipocytokine signaling pathway	*CPT1A, NPY, AGRP*	0.048

BP, biological process; CC, cellular component; MF, molecular function; PWY, metabolic pathway; R_PWY, biochemical reactions and signaling.

One of the biological annotated was the Insulin Signaling and Pathways IGF1 (R_BTA-74752, R_BTA-77387, R_BTA-2428924), with the involvement of *Fibroblast Growth Factor 19* (*FGF19*), *ATPase H+ Transporting V0 Subunit D1* (*ATP6V0D1*), *ATPase H+ Transporting V1 Subunit B2* (*ATP6V1B2*), *Insulin-like Growth Factor 2* (*IGF2*), *Fibroblast Growth Factor 3* (*FGF3*) and *Insulin* (*INS*). These pathways are linked to insulin receptor signaling and IGF1 receptor activation, both of which are critical for energy metabolism and growth (Hakuno and Takahashi, 2018; Al-Massadi et al., 2022; Werner, 2023). The insulin pathway regulates glucose uptake, energy storage, and lipid metabolism (Hakuno and Takahashi, 2018), directly influencing feed efficiency and body weight gain. In cattle, variations in these processes can lead to differences in nutrient utilization, thereby affecting DMI and, consequently, feed efficiency.

The Cascata MAPK (GO:0043410, GO:0030334) was also annotated in the enrichment analyses, involving *Moloney Murine Sarcoma Viral Oncogene* (*MOS*), *LYN Proto-Oncogene, Src Family Tyrosine Kinase* (*LYN*), *Insulin-like Growth Factor 2* (*IGF2*) and *Gasdermin E* (*GSDME*) genes. Processes such as growth, differentiation, and response to environmental stress are regulated by this pathway (Avila et al., 2012; Adhikari and Cullen, 2014; Gonzalez-Garcia et al., 2014; Werner, 2023). The influence of these processes on DMI may be related to how animals respond to their environment, impacting their nutritional requirements and feed intake. Genes such as *LYN* and *IGF2*, which are associated with growth and development (Avila et al., 2012; Pereira et al., 2016; Terakado et al., 2018; Rieger and O’Connor, 2021), further highlight the importance of this pathway in managing energy demands. Another crucial pathway is the PI3K-AKT signaling pathway (R_BTA-1257604, R_BTA-6811558), involving the *FGF19*, *FGF3*, and *Fibroblast Growth Factor 4 (FGF4)*, as well as *INS* genes. The PI3K-AKT pathway is central to cell survival, growth, and metabolism, particularly in insulin response (Hardy et al., 2011; Toschi and Baratta, 2021; Yang et al., 2022). In cattle, this pathway is closely linked to feed efficiency and nutrient metabolism, influencing how animals efficiently convert feed into body mass (Cantalapiedra-Hijar et al., 2018; Toschi and Baratta, 2021; Yang et al., 2022). Variations in genes related to this pathway could alter how energy is allocated for growth, maintenance, and reproduction, thereby affecting DMI.

The Ras signaling pathway (bta04114, bta04015) was also identified, with genes such as *FGF19*, *IGF2*, *FGF3*, and *FGF4* involved in this process. This pathway regulates cell proliferation, differentiation, and survival (Huang et al., 2014; Nies et al., 2016). It can influence growth and metabolism in response to environmental stressors, directly impacting DMI. For instance, cattle exposed to adverse conditions may experience altered metabolic demands, and genes such as *FGF19* and *IGF2* can modulate these responses, leading to changes in feed intake (Mota et al., 2022). The regulation of the actin cytoskeleton (GO:0005824, GO:0008543) was also annotated in the enrichment analyses, involving genes such as *MOS*, *FGF3*, *FGF4*, *IGF2*. This process includes alterations in cellular structure, which are essential for various cellular functions such as growth and mobility (Illescas et al., 2021; Gao and Nakamura, 2022; Dehghanian Reyhan et al., 2023). It may affect muscle development and maintenance, key factors in determining the energy demands of cattle (Dehghanian Reyhan et al., 2023; Arikawa et al., 2024; Sacarrao-Birrento et al., 2024), and consequently, could influence DMI. In the context of DMI, this process may influence how animals metabolize nutrients and convert feed into body mass efficiently, affecting feed intake requirements. The genes and pathways identified for the DMI intercept are central to metabolic processes that regulate growth, energy balance, and nutrient utilization. These biological processes are particularly important for animals raised under variable environmental conditions, such as Nellore cattle, which may impact feed intake and efficiency.

#### 3.2.4 Slope for DMI

For the DMI slope across EG levels, 17 relevant genomic windows were identified (Table 9; Figure 2). These genomic windows are located on ten chromosomes: BTA1 (155.72–156.03 Mb), BTA2 (104.16–104.55 Mb and 104.58–105.27 Mb), BTA5 (15.53–15.88 Mb and 65.97–66.93 Mb), BTA6 (2.32–2.80 Mb), BTA9 (49.80–50.31 Mb), BTA11 (4.85–5.21 Mb, 5.55–5.92 Mb and 100.94–101.52 Mb), BTA13 (41.40–41.97 Mb), BTA14 (22.90–23.31 Mb and 23.33–23.89 Mb), BTA18 (32.19–32.54 Mb and 35.62–36.07 Mb) and BTA29 (48.74–50.54 Mb). In other GWAS studies with Nellore cattle, some of these regions were also associated with DMI (Brunes et al., 2020; Mota et al., 2022). A total of 111 genes were identified within these genomic windows, including 109 protein-coding genes and two snRNAs.

**TABLE 9:** List of the top genomic windows that explained more than 1% of the total direct additive genetic variance ( $\sigma_{a}^{2}$ ) for dry matter intake (DMI - slope).

BTA	Location (Mb)	Genes	$\sigma_{a}^{2}$ (%)
14	22.90–23.31	*XKR4, TMEM68, TGS1, LYN, RPS20, U1, MOS, PLAG1, CHCHD7, SDR16C5, SDR16C6, PENK, U6, BPNT2*	3.55
5	65.97–66.93	*PARPBP, PMCH, IGF1, U6, PAH, ASCL1, U1*	3.33
18	32.19–32.54*	*-*	3.21
13	41.40–41.97	*FOXA2, U6, THBD, CD93*	3.09
2	104.16–104.55	*XRCC5, MARCHF4, SMARCAL1*	2.96
18	34.95–35.60	*LRRC36, TPPP3, ZDHHC1, HSD11B2, ATP6V0D1, AGRP, RIPOR1, CTCF, CARMIL2, ACD, PARD6A, ENKD1, C18H16orf86, GFOD2, RANBP10, TSNAXIP1, CENPT, THAP11, NUTF2, EDC4, NRN1L, PSKH1, PSMB10, LCAT, SLC12A4, DPEP3, DPEP2, DDX28, DUS2, NFATC3, U6*	2.93
2	104.58–105.27	*IGFBP2, IGFBP5, TNP1*	2.87
11	5.55–5.92	*NMS, PDCL3, NPAS2*	2.17
1	155.72–156.03*	*-*	2.09
6	2.32–2.80	*NPY5R, NPY1R, NAF1, U6*	1.94
14	23.33–23.89	*PLAG1, CHCHD7, SDR16C5, SDR16C6, PENK, U6, BPNT2*	1.61
5	15.53–15.88	*NTS, MAGT4C, PARPBP, PMCH, IGF1, U6, PAH, ASCL1, U1*	1.46
29	48.74–50.54	*KCNQ1, TRPM5, TSSC4, TSPAN32, ASCL2, TH, INS, IGF2, TNNT3, LSP1, PRR33, TNNI2, SYT8, CTSD, IFITM10, DUSP8, MOB2, BRSK2, TOLLIP, MUC2, AP2A2*	1.35
11	100.94–101.52	*PRDM12, EXOSC2, ABL1, QRFP, FIBCD1, LAMC3, NUP214, FAM78A, PLPP7*	1.32
9	49.80–50.31	*MCHR2, PRDM13, U6, CCNC, TSTD3, USP45*	1.30
18	35.62–36.07	*ESRP2, PLAG2G15, SLC7A6, SLC7A6OS, PRMT7, SMPD3, ZPF90, CDH3, CDH1*	1.20
11	4.85–5.21	*AFF3*	1.19

BTA: *bos taurus autosome*.

A total of 37.57% of the overall direct additive genetic variance was captured by the relevant genomic regions identified. The genomic window located on BTA14 (22.90–23.31 Mb) explained the largest portion of additive genetic variance, accounting for 3.55%, with 14 annotated genes identified within this window (Table 9). The *XK Related 4* (*XKR4*) gene encodes a protein involved in apoptosis and membrane remodeling (Chakraborty et al., 2024; Song et al., 2024). *XKR4* is expressed in a wide range of tissues, including the nervous system and muscles (Xu P. et al., 2020; Yu et al., 2024). Given that DMI influences muscle growth and energy balance, the role of *XKR4* in muscle-related processes (Edea et al., 2020) may render it significant for energy metabolism, and consequently, for feed intake and utilization under varying environmental conditions. Another relevant gene, *Transmembrane Protein 68* (*TMEM68*), is implicated in lipid metabolism (Edea et al., 2020; Wang et al., 2023; Zeng et al., 2024). Genes involved in lipid metabolism are generally critical for energy storage and utilization (Srivastava et al., 2020). Lipid metabolism plays a pivotal role in feed efficiency by regulating how energy is stored, mobilized, and used by the animal. In more feed-efficient cattle, enhanced lipid oxidation pathways and more effective lipid transport have been observed, leading to greater energy availability for growth and maintenance (Artegoitia et al., 2019; Yang et al., 2023). Additionally, these animals tend to exhibit reduced hepatic lipid synthesis and accumulation, further supporting the association between lipid metabolism and improved nutrient utilization (Taiwo et al., 2022). Altogether, these findings highlight the importance of lipid metabolic pathways in promoting feed efficiency in beef cattle. As lipid metabolism is closely linked to feed efficiency, *TMEM68* may influence the conversion rate of feed into energy, particularly under diverse environmental conditions, thereby influencing total feed intake. Another gene identified was *Pleomorphic Adenoma Gene 1 (PLAG1)*. Its role is involved in regulating growth and development, particularly influencing body size and stature (Hou et al., 2019; Zhang et al., 2022; Pan et al., 2022). The effect of *PLAG1* on growth potentially makes it a critical gene for feed efficiency. Cattle with variants of this gene that promote more efficient growth may exhibit different patterns of DMI, particularly under variable environmental conditions.

The *Coiled-Coil-Helix-Coiled-Coil-Helix Domain Containing 7 (CHCHD7)* gene is involved in mitochondrial function, specifically in maintaining mitochondrial integrity (Li et al., 2020; Yan et al., 2024). This gene is also associated with growth and stature in several species, including cattle (Li et al., 2020; Xu H. et al., 2020; Pan et al., 2022; Kolpakov et al., 2024). Mitochondria are central to energy production, and variations in genes affecting mitochondrial efficiency can influence energy metabolism, thereby impacting how much feed is required to maintain or support growth under different environmental conditions. Another gene, *Proenkephalin (PENK)*, encodes a precursor for enkephalins, which are neuropeptides involved in pain regulation and stress responses (Adhikari et al., 2022; Pierzchała-Koziec and Scanes, 2023). Stress responses can influence appetite and metabolism in cattle (Fernandez-Novo et al., 2020; Sammad et al., 2020; Meneses et al., 2021). Variations in *PENK* may affect how cattle respond to environmental stressors, thereby influencing their feeding behavior and metabolic efficiency.

A total of 42 processes were found to be significantly linked (p-value <0.05) with the slope of DMI (Table 10). One of the key processes identified is signaling by the Insulin Receptor (R-BTA-74752), which is critical for glucose metabolism and overall energy homeostasis. The insulin receptor pathway controls how cells take up glucose from the bloodstream, a process essential for energy production (Pereira et al., 2016; Rieger and O’Connor, 2021; Díaz Del Moral et al., 2022). Genes like *FGF19*, *ATP6V1B2*, and *INS* are involved in this pathway, with *INS* directly regulating nutrient uptake and metabolism (Payankaulam et al., 2019). In the context of DMI slope, these genes may influence how cattle adjust their feed intake in response to energy needs, impacting their efficiency in converting feed into energy under variable environmental conditions.

**TABLE 10:** Significant Gene Ontology (GO) terms and Kyoto Encyclopedia of Genes and Genomes (KEGG) pathway analyses for dry matter intake (DMI - slope).

Category	Terms	Gene symbol	p-value
BP	R-BTA-74752 - Signaling by Insulin receptor	*FGF19, ATP6V1B2, ATP6V0D1, CTSD, FGF3, FGF4, INS*	<0.001
BP	R-BTA-9006934 - Signaling by Receptor Tyrosine Kinases	*FGF19, ATP6V1B2, IGF2, ATP6V0D1, CTSD, FGF3, FGF4, INS*	<0.001
BP	R-BTA-77387 - Insulin receptor recycling	*ATP6V1B2, ATP6V0D1, CTSD, INS*	<0.001
R_PWY	GO:0009887 - Animal organ morphogenesis	*FGF19, E2F4, PHLDA2, FGF3, FGF4*	<0.001
BP	R-BTA-2428928 - IRS-related events triggered by IGF1R	*FGF19, IGF2, FGF3, FGF4*	<0.001
MF	R-BTA-2428924 - IGF1R signaling cascade	*FGF19, IGF2, FGF3, FGF4*	0.001
BP	R-BTA-2404192 - Signaling by Type 1 Insulin-like Growth Factor 1 Receptor (IGF1R)	*FGF19, IGF2, FGF3, FGF4*	0.001
CC	R-BTA-74751 - Insulin receptor signaling cascade	*FGF19, FGF3, FGF4, INS*	0.001
BP	GO:0043410 - Positive regulation of MAPK cascade	*MOS, LYN, IGF2, GSDME, INS*	0.001
BP	GO:0005737 - Cytoplasm	*RIPOR1, BRSK2, TSSC4, FHOD1, IGHMBP2, NCAPG, FGF3, FGF4, HERC5, HERC3, CCND1, TSNAXIP1, E2F4, PHLDA2, LZTS1, HERC6, LYN, TDRD9, DUSP8, NAP1L4, THAP11, MED28, MOS, TPPP3, TH, KIF26A, FGF19, MOB2, RANBP10, ELMO3, LAP3, CARS1, GSDME*	0.003
BP	GO:0001934 - Positive regulation of protein phosphorylation	*CCND1, FGF19, MOB2, FGF3, FGF4*	0.003
BP	R-BTA-1257604 - PIP3 activates AKT signaling	*FGF19, FGF3, PSMB10, FGF4, INS*	0.005
PWY	R-BTA-199418 - Negative regulation of the PI3K/AKT network	*FGF19, FGF3, FGF4, INS*	0.005
MF	R-BTA-6811558 - PI5P, PP2A and IER3 Regulate PI3K/AKT Signaling	*FGF19, FGF3, FGF4, INS*	0.006
MF	R-BTA-9006925 - Intracellular signaling by second messengers	*FGF19, FGF3, PSMB10, FGF4, INS*	0.006
BP	GO:0005104 - Fibroblast growth factor receptor binding	*FGF19, FGF3, FGF4*	0.007
R_PWY	GO:0010628 - Positive regulation of gene expression	*PLAG1, FGF19, CTCF, FGF3, FGF4, INS*	0.007
R_PWY	bta05218 - Melanoma	*CCND1, FGF19, FGF3, FGF4*	0.012
MF	bta04014 - Ras signaling pathway	*FGF19, IGF2, PLD1, FGF3, FGF4, INS*	0.014
MF	GO:0030334 - Regulation of cell migration	*FGF19, PHLDA2, FGF3, FGF4*	0.014
BP	R-BTA-109704 - PI3K Cascade	*FGF19, FGF3, FGF4*	0.016
PWY	R-BTA-112399 - IRS-mediated signaling	*FGF19, FGF3, FGF4*	0.017
BP	GO:0003779 - actin binding	*MAP1B, TNNT3, TNNI2, LSP1, MED28*	0.023
R_PWY	GO:0051781 - Positive regulation of cell division	*IGF2, FGF3, FGF4*	0.025
R_PWY	GO:0045121 - Membrane raft	*LYN, KCNQ1, CTSD, ABCG2*	0.032
BP	bta04010 - MAPK signaling pathway	*FGF19, IGF2, DUSP8, FGF3, FGF4, INS*	0.032
BP	GO:0006006 - Glucose metabolic process	*KCNQ1, IGF2, INS*	0.032
BP	R-BTA-917937 - Iron uptake and transport	*ATP6V1B2, ATP6V0D1, ABCG2*	0.033
MF	GO:0008543 - Fibroblast growth factor receptor signaling pathway	*FGF19, FGF3, FGF4*	0.033
MF	R-BTA-162582 - Signal Transduction	*CPT1A, FGF19, NPY, PENK, ATP6V1B2, IGF2, ATP6V0D1, CTSD, FGF3, PSMB10, FGF4, INS*	0.033
BP	bta04015 - Rap1 signaling pathway	*PARD6A, FGF19, FGF3, FGF4, INS*	0.036
R_PWY	GO:0008083 - Growth factor activity	*FGF19, IGF2, FGF3, FGF4*	0.037
CC	bta04810 - Regulation of actin cytoskeleton	*MOS, FGF19, FGF3, FGF4, INS*	0.037
MF	GO:0008343 - Adult feeding behavior	*NPY, AGRP*	0.038
R_PWY	R-BTA-190236 - Signaling by FGFR	*FGF19, FGF3, FGF4*	0.039
BP	R-BTA-5658623 - FGFRL1 modulation of FGFR1 signaling	*FGF3, FGF4*	0.041
BP	R-BTA-382551 - Transport of small molecules	*SLC9A5, SLC22A18, ATP6V1B2, ATP6V0D1, PSMB10, ABCG2*	0.041
PWY	GO:0016324 - Apical plasma membrane	*PARD6A, KCNQ1, ATP6V1B2, PLD1, ABCG2*	0.043
MF	GO:0005861 - Troponin complex	*TNNT3, TNNI2*	0.047
BP	bta05224 - Breast cancer	*CCND1, FGF19, FGF3, FGF4*	0.049
BP	GO:1902600 - Proton transmembrane transport	*SLC9A5, ATP6V1B2, ATP6V0D1*	0.049
R_PWY	bta05226 - Gastric cancer	*CCND1, FGF19, FGF3, FGF4*	0.049

BP, biological process; CC, cellular component; MF, molecular function; PWY, metabolic pathway; R_PWY, biochemical reactions and signaling.

Similarly, the *Receptor Tyrosine Kinases* (*RTK*) signaling pathway (R-BTA-9006943), which includes genes such as *FGF19*, *FGF3*, and *INS*, is involved in cellular growth, proliferation, and metabolism (Schlessinger, 2000). *RTKs* play a pivotal role in transmitting extracellular signals to the cell’s interior, regulating growth and development processes (Schlessinger, 2000). Variations in these genes could affect how cattle respond to growth-related signals, potentially altering their feed intake based on growth demands in different environments, which could explain variation in the DMI slope.

The Insulin-like Growth Factor 1 Receptor (*IGF1R*) signaling pathway (R-BTA-2428924) also emerged as significant. This pathway is crucial for growth and development, influencing cell growth, differentiation, and survival (Pereira et al., 2016; Rieger and O’Connor, 2021; Díaz Del Moral et al., 2022). Genes like *FGFR3*, *FGFR4*, and *INS* are associated with this process. Given that IGF1R signaling regulates anabolic processes and energy usage, it is plausible that variations in this pathway could influence how efficiently cattle manage their energy resources when environmental conditions fluctuate, thus impacting the slope of DMI.

The *PI3K/AKT* signaling pathway (R-BTA-1257044) is another important metabolic pathway linked to the regulation of growth and survival, particularly under conditions of nutrient stress (Edinger, 2007). This pathway includes genes like *PI3K*, *AKT1*, and *PSMB10*, which are involved in cell survival, proliferation, and glucose metabolism (Edinger and Thompson, 2004; Edinger, 2007; Wu et al., 2016). As this pathway integrates signals related to nutrient availability, it likely plays a role in determining how cattle adjust their intake to optimize growth and energy storage under variable conditions, affecting the slope of DMI.

Additionally, pathways related to Fibroblast Growth Factor Receptor (FGFR) signaling (GO-0005104) and Ras signaling (GO-0043404) were highlighted. These pathways involve genes like *FGFR1*, *FGFR3*, and *FGF19*, which are critical for cell proliferation, differentiation, and metabolism (Itoh and Ornitz, 2004; Itoh, 2007). The Ras pathway is central in transmitting signals that regulate cellular growth and energy use (Huang et al., 2014; Nies et al., 2016). Variations in these genes might influence how cattle balance their growth and metabolic processes in response to environmental changes, impacting their feed efficiency and DMI slope.

Moreover, cytoplasmic processes (GO:0005737) and positive regulation of protein phosphorylation (GO:0001934) are involved in cellular signaling and metabolic regulation (Hüttemann et al., 2007; Hermann et al., 2008; Humphrey et al., 2015; Zhu and Thompson, 2019). Genes such as *INS*, *FGFR4*, and *PSMB10* play crucial roles in modulating these processes (Hüttemann et al., 2007; Zhu and Thompson, 2019). These pathways could influence the efficiency of nutrient metabolism and energy utilization, thereby affecting how animals adapt their feed intake to varying environmental conditions, which in turn affects the DMI slope.

In summary, the processes identified in the enrichment analysis, particularly those involved in insulin signaling, growth factor signaling, and cellular metabolism, suggest a strong connection between the regulation of energy balance and the slope of DMI in Nellore cattle. The genes involved in these pathways, such as *FGF19*, *FGFR3*, and *INS*, are likely to affect the cattle’s ability to adjust their feed intake in response to changing environmental conditions, influencing their overall efficiency and adaptability. This genomic information can provide a foundation for improving feed efficiency and productivity in livestock through targeted breeding strategies.

### 3.3 Functional networks for RFI

**FIGURE 3:**
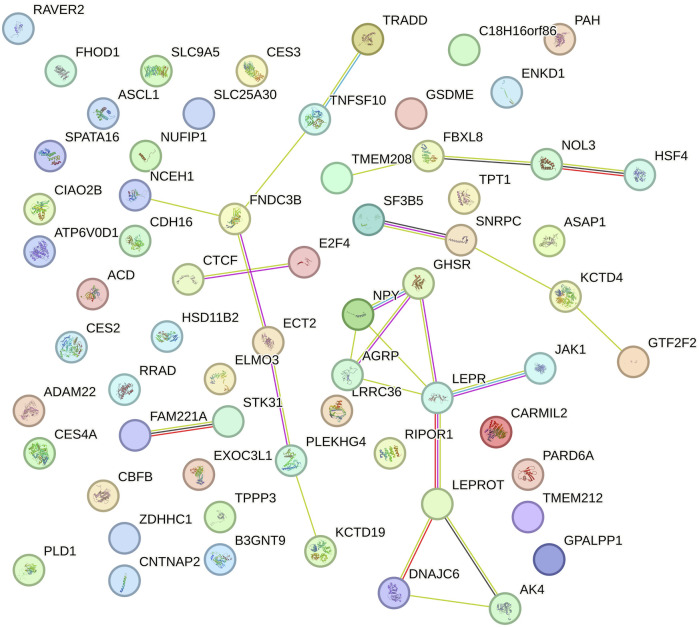
Functional network of genes identified in the genomic windows that explained more than 1% of the total direct additive genetic variance of the intercept for residual feed intake (RFI) in Nellore cattle. Each node represents a gene, while the lines connecting the nodes indicate known functional interactions or associations between these genes. The different colors of the nodes and lines indicate distinct types of interactions or classifications of biological functions, based on the network analysis.

The functional networks of the candidate genes identified for RFI in Nellore cattle showed significant changes in connectivity and the central role of certain genes between the intercept and slope. These results illustrate the GxE interaction, highlighting how different gene networks are mobilized depending on the environmental influence on the phenotype. In the network related to the intercept (Figure 3), genes such as *LEPR*, *LEPROT*, *NPY*, *GHSR*, and *AGRP* exhibited high connectivity, forming a functional core with several interactions. These genes are closely associated with appetite regulation and energy metabolism, such as *GHSR* and *LEPR*, which are known for their roles in regulating feeding behavior and energy efficiency, suggesting that central metabolic pathways are key determinants in the baseline genetic variation of RFI. Additionally, several smaller sub-networks with fewer connections were identified, indicating that these genes may be linked to more specific processes or sub-functions within the regulation of RFI.

**FIGURE 4:**
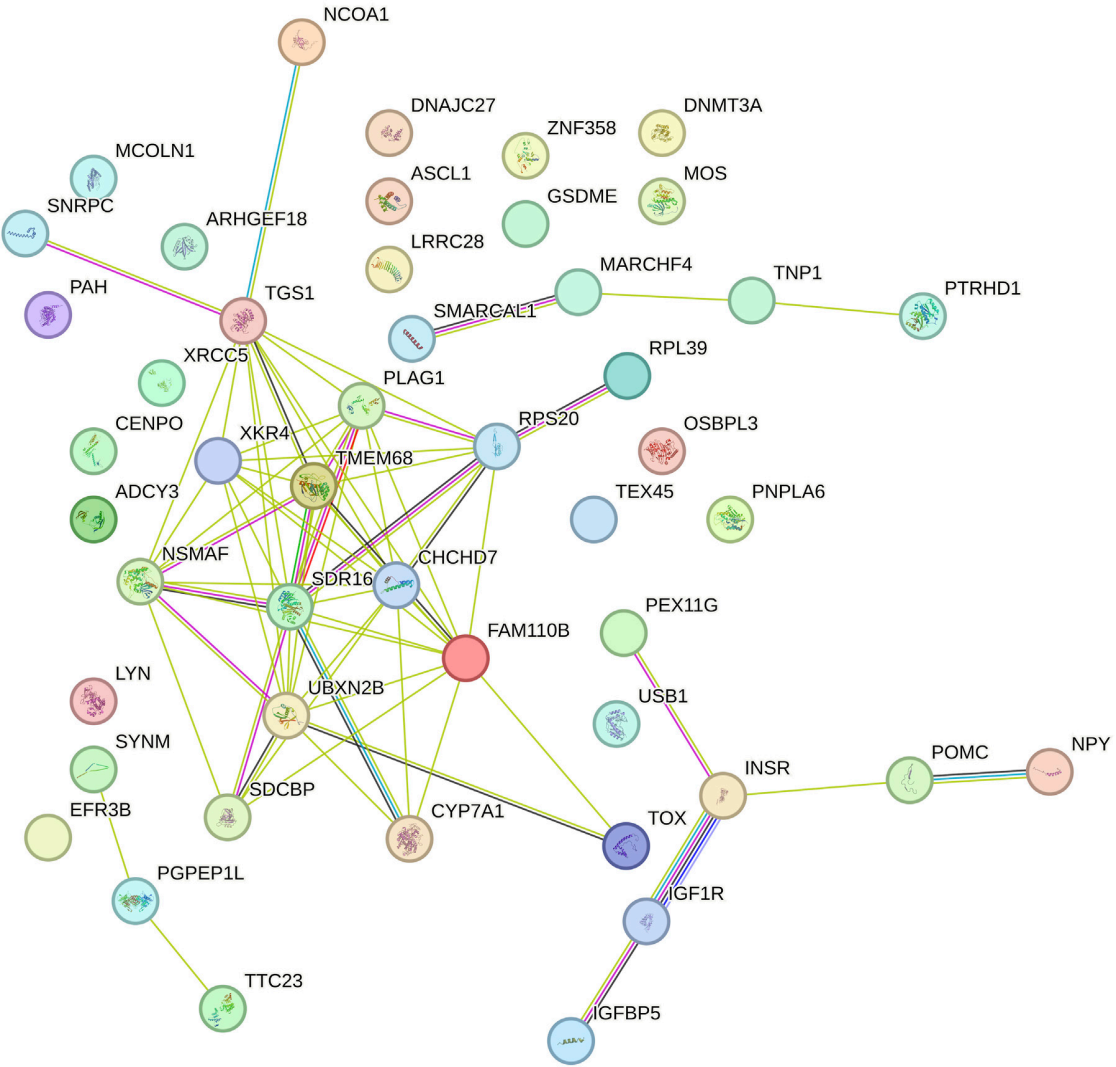
Functional network of genes identified in the genomic windows that explained more than 1% of the total direct additive genetic variance of the slope for residual feed intake (RFI) in Nellore cattle. Each node represents a gene, while the lines connecting the nodes indicate known functional interactions or associations between these genes. The different colors of the nodes and lines indicate distinct types of interactions or classifications of biological functions, based on the network analysis.

In contrast, in the network related to the slope of RFI (Figure 4), there was a shift in the most connected genes. Genes such as *TMEM68*, *XKR4*, *CHCHD7*, *RPS20*, *PLAG1*, and *FAM110B* emerged as central, exhibiting multiple interactions with other genes. This indicates that distinct genetic mechanisms may be involved in the variation of RFI over time, with feed intake regulation being mediated by different genetic pathways. The interaction between genes related to energy metabolism and growth is evidenced by the connection of *INSR* with *IGF1R* and *IGFBP5*, which are fundamental to insulin signaling. Additionally, *NPY* and *POMC* indicate the influence of appetite control pathways on feed efficiency. The network also includes genes such as *CYP7A1* and *SDR16C5*, involved in lipid metabolism, that interact with *CHCHD7* and *UBXN2B*, suggesting a role in lipid metabolism in the variation of the slope of RFI. Genes associated with mitochondrial function, such as *TMEM68* and *CHCHD7*, emphasize the importance of cellular health in feed efficiency. Finally, small subnetworks formed by genes like *PTRH1*, *DNMT3A*, and *MOS* indicate potential more specific functions, such as epigenetic regulation and response to stressors. This complex network highlights the interconnection of multiple biological processes that influence feed efficiency under different environmental conditions.

### 3.4 Functional gene networks for DMI

**FIGURE 5:**
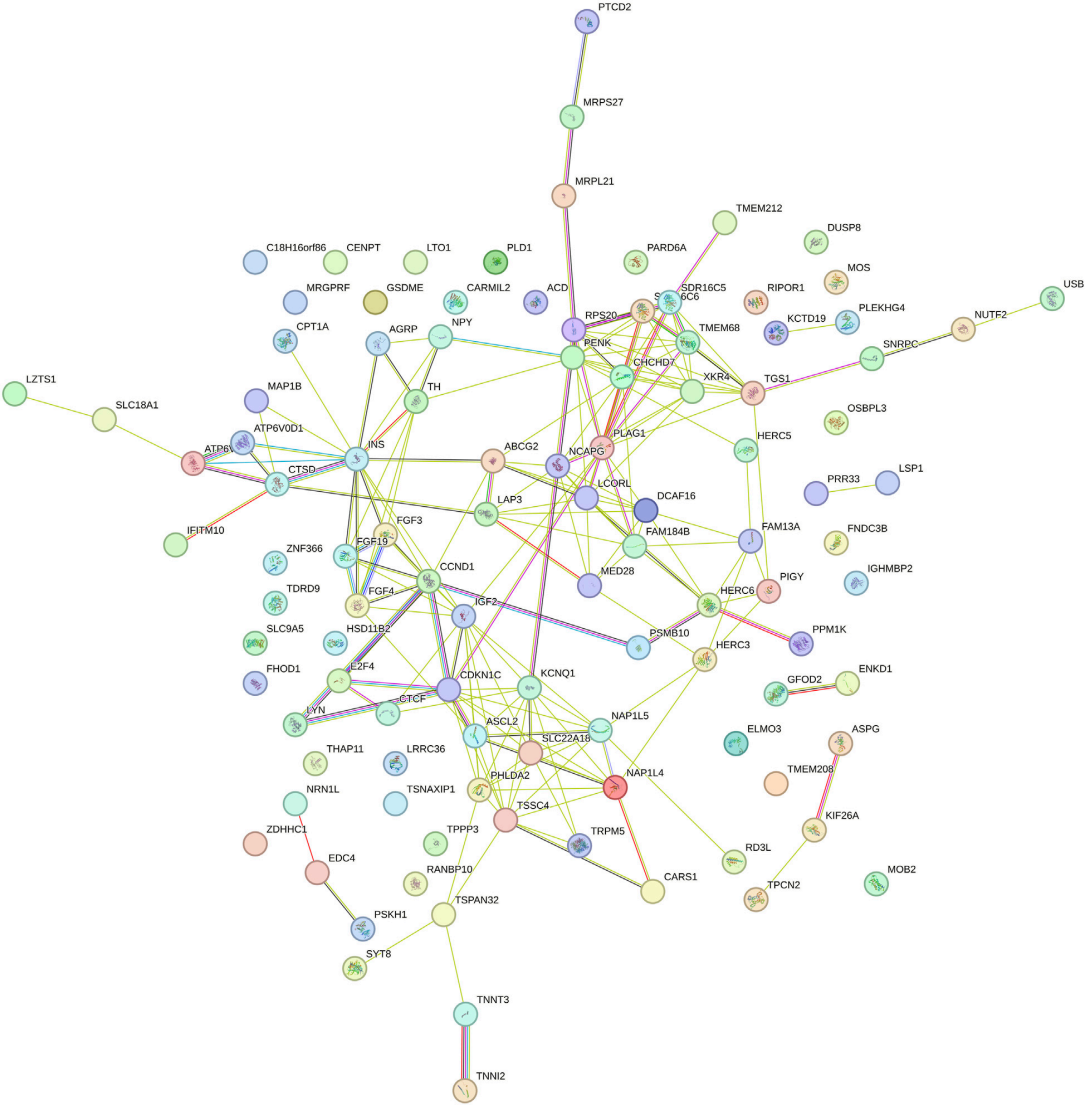
Functional network of genes identified in the genomic windows that explained more than 1% of the total direct additive genetic variance of the intercept for dry matter intake (DMI) in Nellore cattle. Each node represents a gene, while the lines connecting the nodes indicate known functional interactions or associations between these genes. The different colors of the nodes and lines indicate distinct types of interactions or classifications of biological functions, based on the network analysis.

**FIGURE 6:**
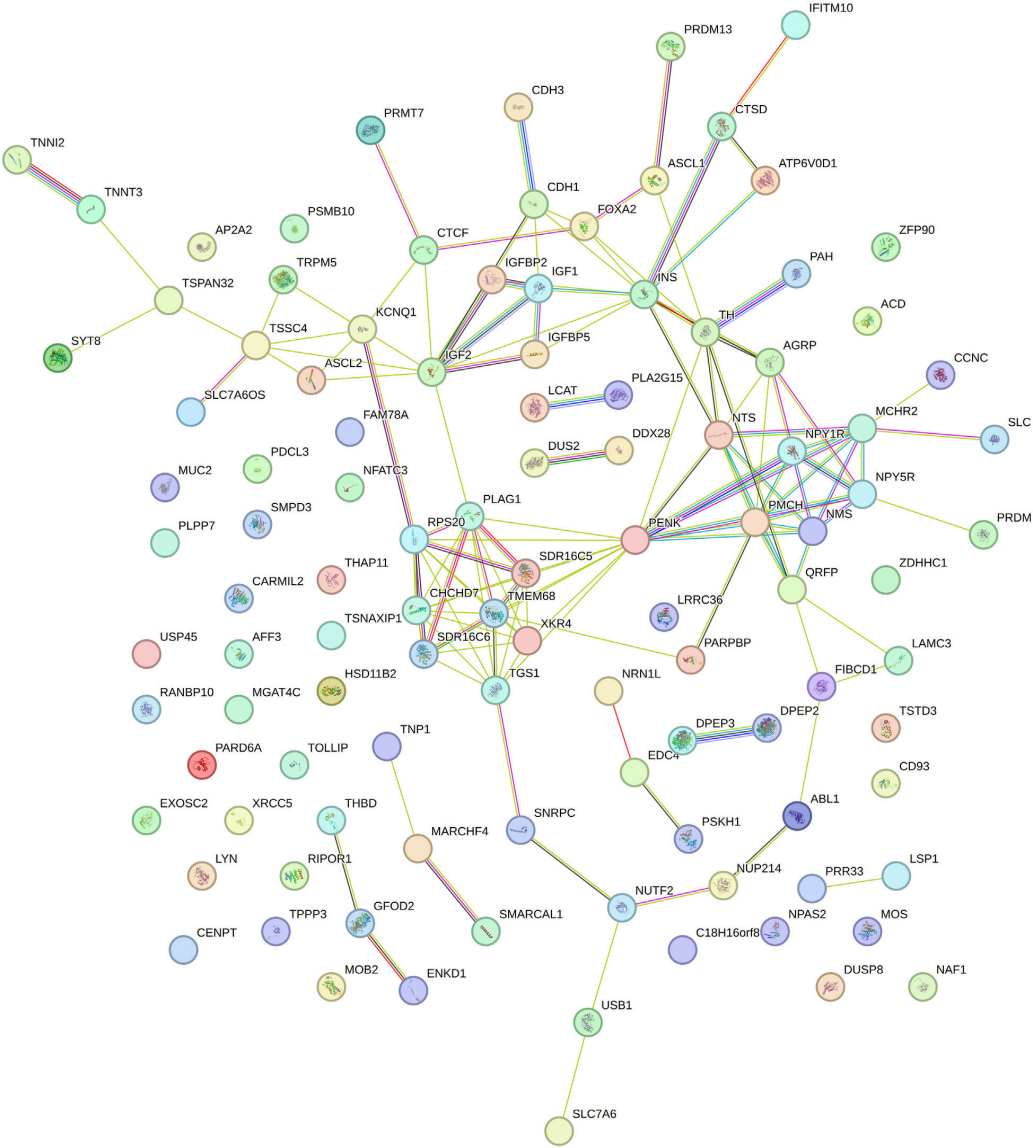
Functional network of genes identified in the genomic windows that explained more than 1% of the total direct additive genetic variance of the slope for dry matter intake (DMI) in Nellore cattle. Each node represents a gene, while the lines connecting the nodes indicate known functional interactions or associations between these genes. The different colors of the nodes and lines indicate distinct types of interactions or classifications of biological functions, based on the network analysis.

Figures 5 (intercept), Figure 6 (slope) present the functional gene networks identified based on the candidate genes identified for DMI in Nellore cattle. In Figure 5, the functional network consists of a dense web of interactions among genes, suggesting strong basal genetic regulation for DMI under controlled environments. Genes such as *PLAG1*, *IGF2*, *CHCHD7*, *CCND1*, and *NCAPG* show centrality, with several direct and indirect connections, indicating their crucial role in regulating this trait. The interactions among these genes stand out as responsible for the genetic architecture of the phenotype in an average environment, with particular emphasis on *NCAPG*, a gene known for its association with growth traits and feed efficiency in cattle (Takasuga, 2015).

Figure 6 represents the genetic modulation in response to the environment in which a complex interaction between central and peripheral genes is observed, highlighting pathways associated with appetite regulation and energy metabolism. Genes such as *IGF2*, *INS*, *PLAG1*, and *PMCH* are strongly connected to other genes related to energy homeostasis, such as *IGFBP5* and *ASCL2*, suggesting their involvement in growth regulation and response to changes in feed intake over time. The gene *NPY1R*, centralized in the network, reinforces its function in appetite regulation and variation of DMI, while less connected sub-networks, such as those involving the *TNNI2* and *SYT8* genes, may indicate specialized functions. The different colors in the connections between genes suggest varied gene interactions, potentially correlated with environmental and dietary factors. The gene *PENK*, which encodes precursors of enkephalins, stands out for its influence on neural signaling and appetite control, suggesting a crucial role in modulating feed intake and feeding behavior, thereby forming, along with the other genes, a complex regulatory network that affects the slope of DMI. Therefore, the differences observed between Figures 5, 6 clearly demonstrate the plasticity of the genetic network in response to environmental changes. While some genes maintain central importance in both contexts, others emerge as key players in genetic modulation in the face of environmental variations, highlighting the role of GxE in regulating DMI in Nellore cattle.

### 3.5 SNP effects by environmental gradient

**FIGURE 7:**
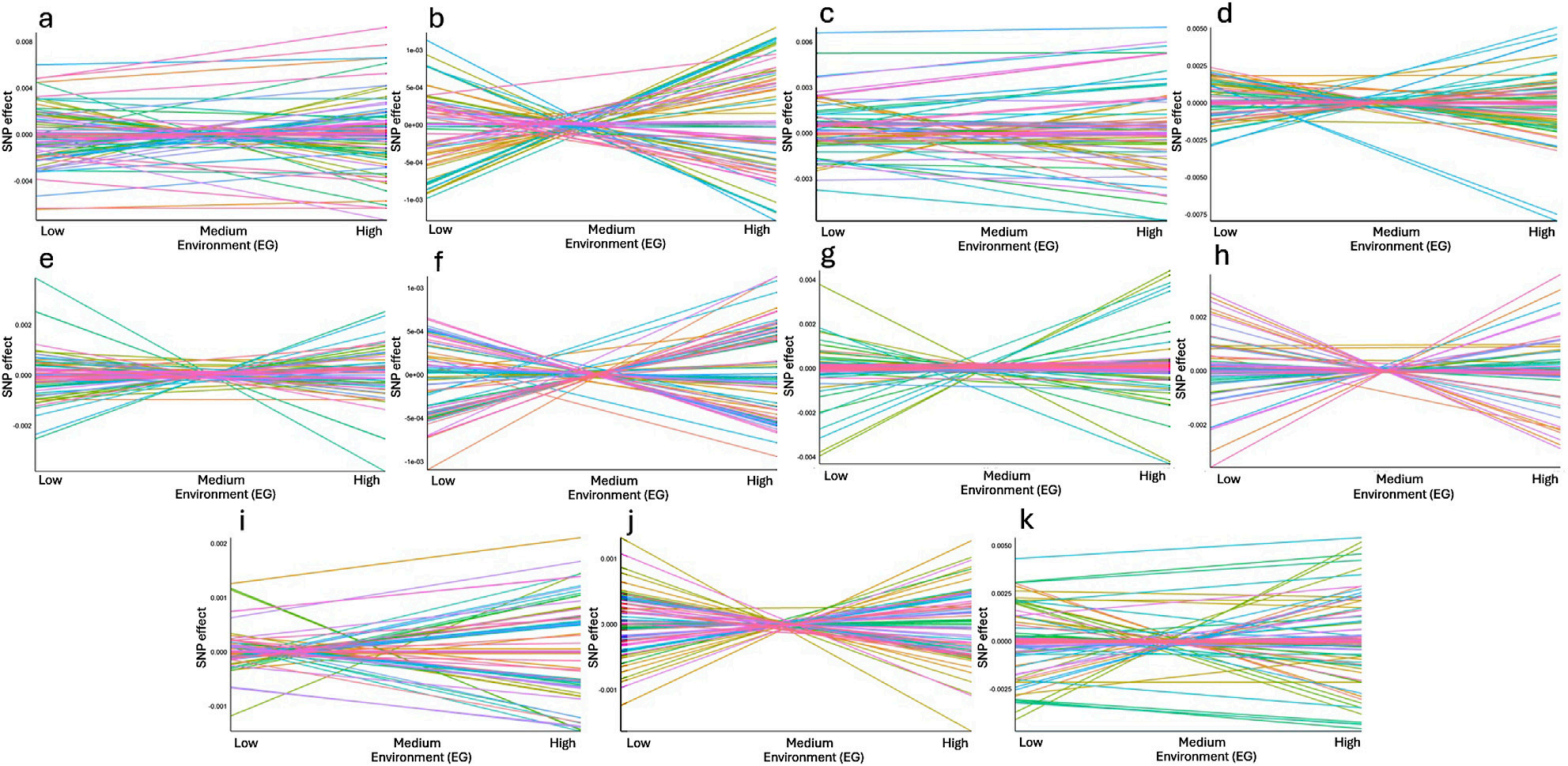
Reaction norms for the effects of all 100 Single Nucleotide Polymorphisms (SNP) comprising the genomic windows **(a-k)** that explained at least 1% of the total additive genetic variance associated with residual feed intake (RFI) in Nellore cattle across low, medium, and high environmental gradients (EG). The graphs are presented in order of the magnitude of additive genetic variance explained by each genomic window, as shown in Table 5, allowing for comparison of SNP impacts under different environmental conditions (EG). Each color of the lines represents a different SNP. Chromosomes and regions: **(a)** BTA4: 70.83–71.85 Mb; **(b)** BTA14: 24.39–24.91 Mb; **(c)** BTA5: 66.51–67.03 Mb; **(d)** BTA21: 7.35–8.15 Mb; **(e)** BTA2: 104.16–104.55 Mb; **(f)** BTA14: 22.99–23.45 Mb; **(g)** BTA2: 104.65–105.41 Mb; **(h)** BTA7: 16.07–16.44 Mb; **(i)** BTA14: 24.91–25.43 Mb; **(j)** BTA14: 22.61–22.99 Mb; **(k)** BTA11: 74.02–74.67 Mb.

The graphs presented in Figure 7 demonstrate the reaction norms of 100 SNPs within the relevant genomic windows (panels “a” to “k”) associated with RFI in Nellore cattle. The effects of the SNPs are plotted across low, medium, and high EG, allowing for the visualization of GxE interactions. The genomic windows are ordered according to their relevance, providing a comparative view of the environmental sensitivity of the SNPs within each genomic window.

SNP-environment interactions are evident in certain genomic windows. Panels such as “b”, “d”, “e”, “f”, “g”, “h” and “j” (BTA14: 24.39–24.91 Mb; BTA21: 7.35–8.15 Mb; BTA2: 104.16–104.55 Mb; BTA14: 22.99–23.45 Mb; BTA2: 104.65–105.41 Mb; BTA7: 16.07–16.44 Mb and BTA14: 22.61–22.99 Mb, respectively) reveal variability in SNP effects as the environment shifts from low to high environmental conditions (extremes), highlighting the presence of GxE interactions (Figure 7). In these cases, the SNPs show differentiated effects depending on environmental conditions, with some alleles exhibiting higher or lower effects as the environmental gradient changes. This suggests that these genomic regions may harbor genes that are particularly sensitive to environmental factors affecting RFI. On the other hand, some genomic windows, such as those located in BTA4: 70.83–71.85 Mb; BTA5: 66.51–67.03 Mb; BTA14: 24.91–25.43 Mb and BTA11: 74.02–74.67 Mb illustrated in panels “a”, “c”, “i”, and “k”, respectively, show relatively more stable SNP effects across the EG (Figure 7). These SNPs appear to be less affected by environmental variation, indicating they may play a more consistent role in RFI across different environments. The greater stability observed in these windows may make them valuable targets for selection when a robust genetic response across environments is desired.

An important pattern observed in several graphs is the crossing of reaction norms, where the effects of SNPs change not only in magnitude but also in direction as the environmental conditions shifts. This highlights the complexity of GxE interactions. The crossed reaction norms underscore the need to consider the environmental context when selecting animals for traits related to RFI, as certain alleles may be beneficial only under specific conditions. Genomic windows with more pronounced changes in SNP effects likely capture a larger share of the genetic variability linked to environmental response. These windows are of particular interest for future research, as they may contain key genes that influence the adaptability of feed efficiency to environmental changes. Identifying these SNPs could lead to more precise genetic selection strategies, improving cattle resilience and performance across different environments.

**FIGURE 8:**
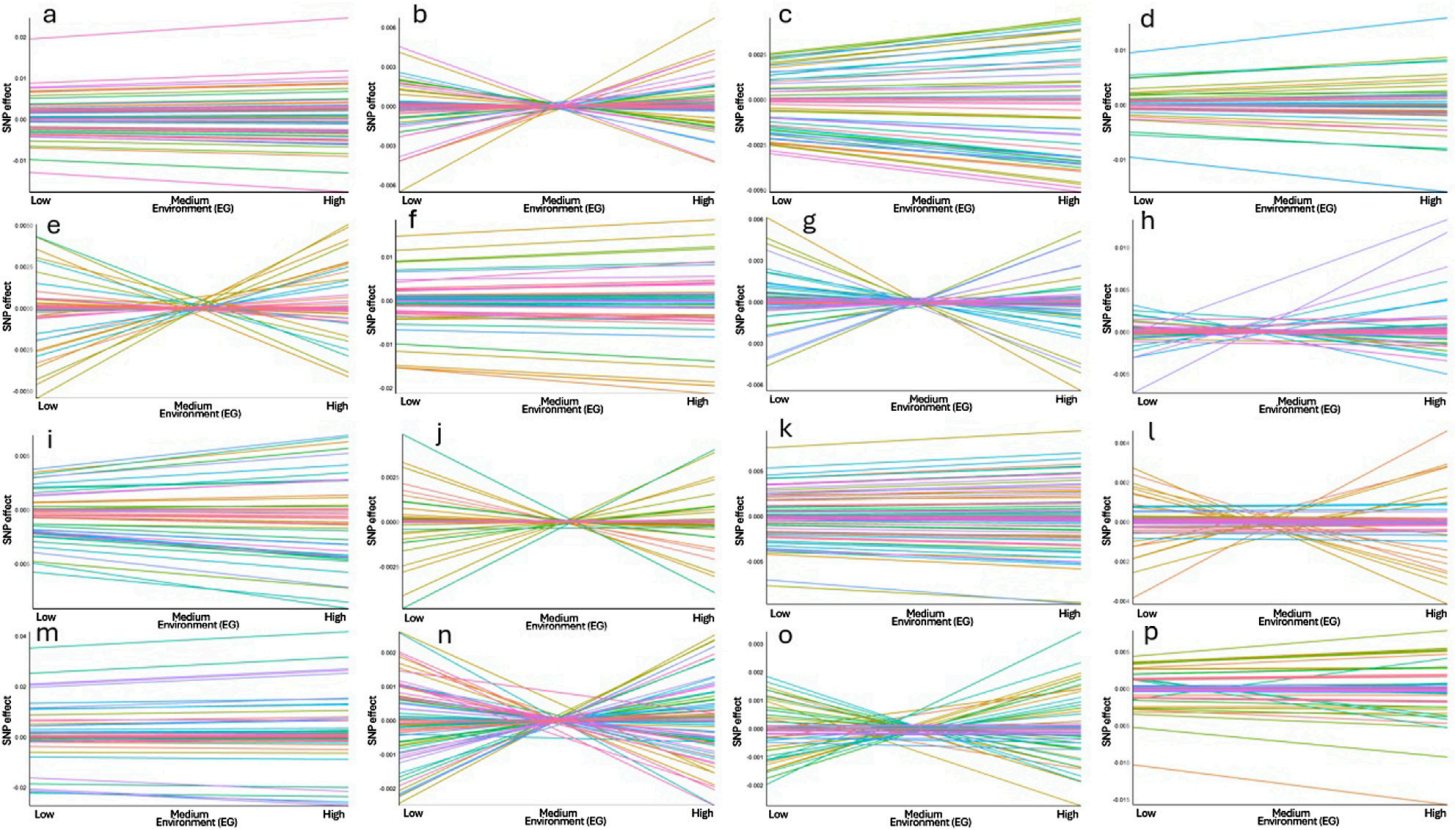
Reaction norms for the effects of all 100 Single Nucleotide Polymorphisms (SNP) comprising each genomic window **(a-p)** that explain at least 1% of the additive genetic variance associated with dry matter intake (DMI) in Nellore cattle across low, medium, and high environmental gradients (EG). The graphs are presented in order of the magnitude of additive genetic variance explained by each genomic window, as shown in Table 9, allowing for comparison of SNP impacts under different environmental conditions (EG). Each color of the lines represents a different SNP. Chromosomes and regions: **(a)** BTA14: 22.90–23.31 Mb; **(b)** BTA5: 65.97–66.93 Mb; **(c)** BTA18: 32.19–32.54 Mb; **(d)** BTA13: 41.40–41.97 Mb; **(e)** BTA2: 104.16–104.55 Mb; **(f)** BTA18: 34.95–35.60 Mb; **(g)** BTA2: 104.58–105.27 Mb; **(H)** BTA11: 5.55–5.92 Mb; **(i)** BTA11: 155.72–156.03 Mb; **(j)** BTA6: 2.32–2.80 Mb; **(k)** BTA14: 23.33–23.89 Mb; **(l)** BTA5: 15.53–15.88 Mb; **(m)** BTA29: 48.74–50.54 Mb; **(n)** BTA11: 100.94–101.52 Mb; **(o)** BTA9: 49.80–50.31 Mb; **(p)** BTA18: 35.62–36.07 Mb.

The effects of SNPs located in the genomic regions associated with DMI in Nellore cattle are shown in Figure 8. Similar to what was observed for RFI, certain genomic regions exhibited interactions between SNPs and the environment. Panels such as “b” (BTA5: 65.97–66.93 Mb), “e” (BTA2: 104.16–104.55 Mb), “g” (BTA2: 104.58–105.27 Mb), “j” (BTA6: 2.32–2.80 Mb), “l” (BTA5: 15.53–15.88 Mb), “n” (BTA11: 100.94–101.52 Mb) and “o” (BTA9: 49.80–50.31 Mb) highlight the variation in SNP effects as the EG shifts from low to high (Figure 8). Furthermore, the crossing of reaction norms was also observed, indicating that the effects of SNPs not only vary in magnitude but also change direction with environmental alterations, suggesting that these genomic regions may harbor genes that are highly sensitive to environmental factors. In contrast, some genomic windows, such as those represented in panels “a” (BTA14: 22.90–23.31 Mb), “c” (BTA18: 32.19–32.54 Mb), “d” (BTA13: 41.40–41.97 Mb), “f” (BTA18: 34.95–35.60 Mb), “i” (BTA11: 155.72–156.03 Mb), “k” (BTA14: 23.33–23.89 Mb), “m” (BTA29: 48.74–50.54 Mb) and “p” (BTA18: 35.62–36.07 Mb) show more consistent SNP effects across EG (Figure 8), indicating that these SNPs are less influenced by environmental variations and may play a more stable role in DMI across different environments.

The variation in SNP effects across EG suggests that breeding programs to improve RFI and DMI should consider GxE interactions. SNPs that exhibit significant positive effects in low environmental gradients may not perform similarly in high gradients, which could impact the genomic selection efficiency of cattle in diverse environments. By identifying SNPs that maintain stable effects across different environments or that are advantageous under specific conditions, breeding strategies can be tailored to optimize feed efficiency. Understanding the genetic architecture of these traits in relation to environmental variation will be crucial for enhancing feed efficiency and sustainability in cattle production, especially considering the increasing challenges posed by climatic variability.

### 3.6 Reaction norms to GEBV for RFI

**FIGURE 9:**
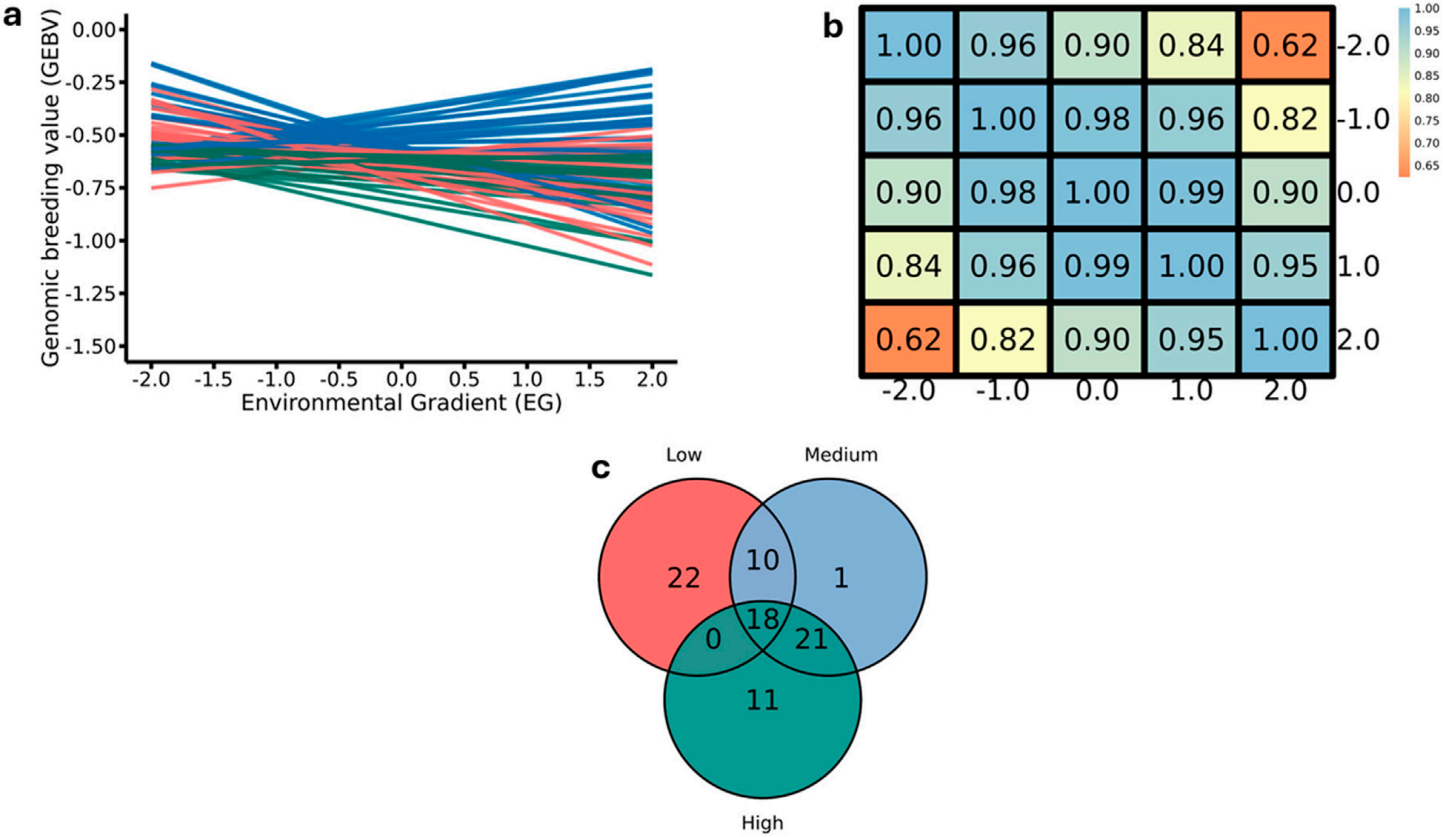
Reaction norms for residual feed intake (RFI) **(a)** Pearson correlation for genomic estimated breeding values (GEBVs) **(b)** and the number of common and specific sires with offspring in the environmental classes for RFI **(c)** considering the 50 sires with the highest number of progeny and top-ranked by GEBV in the moderate environmental gradient (EG = 0.0). The colors of the lines in panel “a” represent the EG, green for medium, red for high and blue for low.

Figure 9 provides a comprehensive analysis of RFI, revealing the complexity of the interaction between GEBVs and EG. In panel “a”, the reaction norms for RFI indicate considerable variation in GEBVs across the EG. Different inclinations suggest that some individuals respond more to environmental variations, while others maintain stable performance, reflecting genetic plasticity. This suggests that the phenotypic response to feed efficiency depends on environmental conditions, emphasizing the importance of considering phenotypic plasticity in genetic improvement strategies.

In panel “b”, Pearson’s correlation analysis highlights the similarity between GEBV values in different EGs, with most correlations exceeding 0.80 (Figure 9). This finding suggests that, while variations exist, sires with high GEBVs tend to maintain their ranking across environments, indicating consistency in the expression of the RFI. However, the lowest correlation (0.62) at the extremes (low and high) of the EG implies that environmental factors may more intensely influence feed efficiency under more divergent environmental conditions, which warrants attention in future selection programs.

Panel “c” illustrates the intersection of sires classified into different EG levels (Figure 9). The presence of 18 sires that stand out across all EG indicates that these individuals possess a robust genetic profile that translates into stable performance across varying environmental conditions. However, the significant number of sires exclusive to one environment (22 in the low EG and 11 in the high EG) suggests reduced precision in selection when contrasting environments are considered. These results provide a foundation for implementing genetic improvement programs aimed at sustainability and productivity, where GxE is considered crucial for optimizing sire selection and maximizing feed performance under diverse environmental conditions.

### 3.7 Reaction norm to GEBV for DMI

**FIGURE 10:**
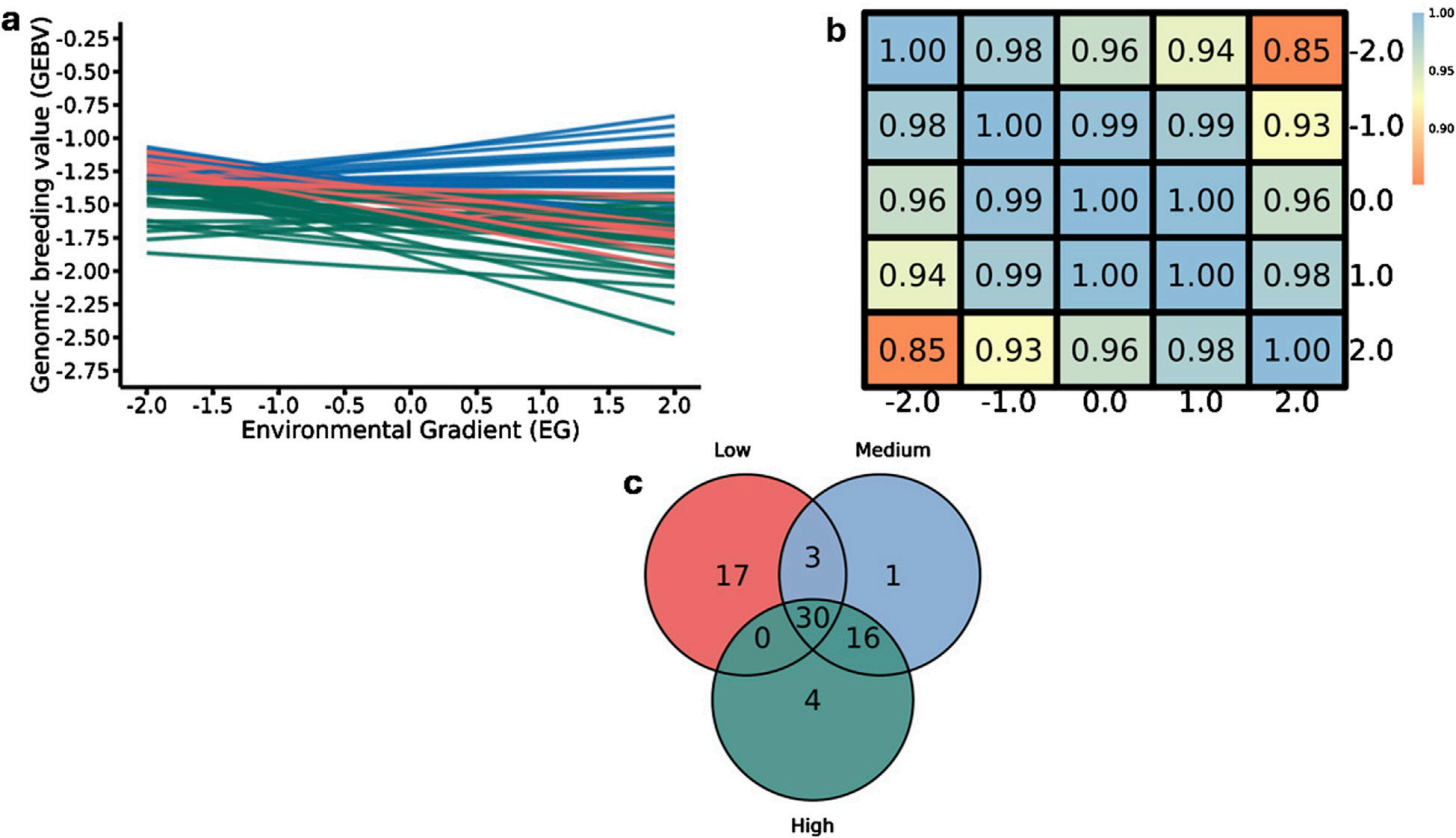
Reaction norms for dry matter intake (DMI) **(a)** Pearson correlation for genomic estimated breeding values (GEBVs) **(b)** and the number of common and specific sires with offspring in the environmental classes for DMI **(c)** considering the 50 sires with the highest number of progeny and top-ranked by GEBV in the moderate environmental gradient (EG = 0.0). The colors of the lines in panel “a” represent the EG, green for medium, red for high and blue for low.

Figure 10 presents the results of the relationships between GEBVs and EG for DMI. In panel “a”, the reaction norms illustrate the variation in GEBVs across the environmental gradient. The sires show different response patterns to environmental changes, with some animals exhibiting increasing GEBVs along the EG (upward lines), while others show a decrease (downward lines). Like for RFI, this indicates heterogeneity in the genetic response to the environment for DMI, highlighting the presence of GxE, as different sires perform variably across different EG.

The Pearson correlation matrix of GEBVs across different EGs for DMI (Figure 10, panel “b”) presents correlations that exceed 0.90, demonstrating a strong consistency in the sires’ GEBV rankings across EG levels. The decrease in the correlation (0.85) at the most different EGs (low and high) underscores a potential variability in DMI responses among sires, even if that is low. Higher Pearson correlations for DMI compared to RFI can be explained by the greater relative stability of the SNPs’ effects present in the genomic windows that explain more additive genetic variance (Figure 8), thus reflecting the observed behavior in the GEBVs. The intersection of sires classified across low, medium, and high EG levels is shown in Figure 10, panel “c”. The presence of 30 sires consistently ranked high across all environments, demonstrating genetic robustness under different EG. However, 17 sires were exclusive to the low EG, and four sires were unique to the high EG, suggesting that these animals may be better adapted to specific environmental conditions. Only three sires stood out in both low and medium EG, while one sire was shared between medium and high EG.

## 4 Conclusion

This study identified key genomic regions associated with RFI and DMI in Nellore cattle, providing significant insights into the genetic background of feed efficiency traits across environmental gradients. For RFI, the intercept network pointed to biological processes crucial for appetite regulation and energy metabolism, emphasizing their role in the genetic variation of RFI in the average environment. The slope network shifted focus to distinct genetic mechanisms influencing RFI variation across EG, including lipid metabolism and mitochondrial function. In the context of DMI, the intercept network featured processes involved in growth regulation, cellular proliferation, and energy metabolism, while the slope network emphasized pathways associated with appetite regulation and energy homeostasis. These findings underscore the adaptability of genetic networks in response to EG influences and highlight the importance of understanding these biological processes, which will be crucial for developing targeted breeding strategies to enhance feed efficiency in Nellore cattle, contributing to improved livestock production and sustainability.

## Data Availability

The data analyzed in this study were obtained from the National Association of Breeders and Researchers (ANCP). The phenotypic and genotypic information was provided to the authors for academic research purposes only. The following restrictions apply: the dataset is not publicly available and its use requires formal authorization. Requests to access these datasets should be directed to Dr. João Carlos G. Giffoni Filho, President of ANCP (email: presidencia@ancp.org.br).
