# Supplementary material for: Genome-wide association study for feed efficiency indicator traits in Nellore cattle considering genotype-by-environment interactions
Source: Front Genet. 2025 Jun 2;16:1539056. doi: 10.3389/fgene.2025.1539056 (PMC12171144; doi:10.3389/fgene.2025.1539056)
Supplement: Supplementary file 1 [file Table1.docx]

| Farm | Region | Köppen-Geiger Climate | Climate Definition | No of Animals |
| --- | --- | --- | --- | --- |
| 1 | Central-West | Aw | A = Tropical; w = Dry Winter | 3,795 |
| 2 | Southeast | Cwa | C = Temperate Humid; w = Dry Winter; a = Hot Summer | 5,282 |
| 3 | Central-West | Aw | A = Tropical; w = Dry Winter | 628 |
| 4 | Southeast | Cwa | C = Temperate Humid; w = Dry Winter; a = Hot Summer | 135 |
| 5 | Central-West | Aw | A = Tropical; w = Dry Winter | 105 |
| 6 | Southeast | Cwa | C = Temperate Humid; w = Dry Winter; a = Hot Summer | 38 |
| 7 | Central-West | Aw | A = Tropical; w = Dry Winter | 1,324 |
| 8 | Northeast | Af | A = Tropical; f = No Dry Season (rain all year round) | 324 |
| 9 | Northeast | Af | A = Tropical; f = No Dry Season (rain all year round) | 2,569 |
| 10 | Southeast | Aw | A = Tropical; w = Dry Winter | 25 |
| 11 | Central-West | Aw | A = Tropical; w = Dry Winter | 588 |
| 12 | Southeast | Aw | A = Tropical; w = Dry Winter | 873 |
| 13 | Central-West | Aw | A = Tropical; w = Dry Winter | 36 |
| 14 | Southeast | Aw | A = Tropical; w = Dry Winter | 361 |
| 15 | Central-West | Aw | A = Tropical; w = Dry Winter | 2,352 |
| 16 | Central-West | Aw | A = Tropical; w = Dry Winter | 212 |
| 17 | Central-West | Aw | A = Tropical; w = Dry Winter | 943 |
| 18 | Southeast | Aw | A = Tropical; w = Dry Winter | 576 |
| 19 | Central-West | Aw | A = Tropical; w = Dry Winter | 322 |
| 20 | North | Af | A = Tropical; f = No Dry Season (rain all year round) | 267 |
| 21 | Central-West | Aw | A = Tropical; w = Dry Winter | 1,292 |
| 22 | Northeast | Af | A = Tropical; f = No Dry Season (rain all year round) | 701 |
| 23 | Central-West | Aw | A = Tropical; w = Dry Winter | 101 |
| 24 | Southeast | Aw | A = Tropical; w = Dry Winter | 219 |
| 25 | Southeast | Aw | A = Tropical; w = Dry Winter | 102 |

Table S1. Environmental characterization of the farms where feed efficiency trials were conducted: region, climate, and number of animals.
